# *Squamanitaceae* and three new species of *Squamanita* parasitic on *Amanita* basidiomes

**DOI:** 10.1186/s43008-021-00057-z

**Published:** 2021-03-03

**Authors:** Jian-Wei Liu, Zai-Wei Ge, Egon Horak, Alfredo Vizzini, Roy E. Halling, Chun-Lei Pan, Zhu L. Yang

**Affiliations:** 1 Yunnan Key Laboratory for Fungal Diversity and Green Development, Kunming Institute of Botany, Chinese Academy of Sciences, Kunming, 650201 Yunnan China; 2 The Germplasm Bank of Wild Species, Kunming Institute of Botany, Chinese Academy of Sciences, 132 Lanhei Road, Kunming, 650201 Yunnan People’s Republic of China; 3 CAS Key Laboratory for Plant Diversity and Biogeography of East Asia, Kunming Institute of Botany, Chinese Academy of Sciences, Kunming, 650201 Yunnan China; 4 Schlossfeld 17, A-6020 Innsbruck, Austria; 5 Department of Life Sciences and Systems Biology, University of Torino and Institute for Sustainable Plant Protection (IPSP-SS Turin), C.N.R, Viale P.A. Mattioli, 25, I-10125 Torino, Italy; 6 Institute of Systematic Botany, New York Botanical Garden, 2900 Southern Blvd., Bronx, NY 10458-5126 USA; 7 Mudanjiang Branch of Heilongjiang Academy of Agricultural Sciences, Mudanjiang, 157041 Heilongjiang China

**Keywords:** *Amanita*, Mycoparasitic fungi, *Squamanita*, Host preference, Three new taxa

## Abstract

The systematic position of the enigmatically mycoparasitic genus *Squamanita* (*Agaricales*, *Basidiomycota*) together with *Cystoderma*, *Phaeolepiota*, *Floccularia*, and *Leucopholiota* is largely unknown. Recently they were recognized as *Squamanitaceae*, but previous studies used few DNA markers from a restricted sample of taxa from the family and lacked a formal taxonomic treatment. In this study, with newly generated sequences of the type of the genus *Squamanita*, *S. schreieri*, and several additional species of the family, the phylogeny is reinvestigated with a concatenated (18S-5.8S-nrLSU-RPB2-TEF1*-*α) dataset. This study reveals that *Cystoderma*, *Phaeolepiota*, *Squamanita*, *Floccularia*, and *Leucopholiota* are a monophyletic clade with strong statistical support in Bayesian analysis and form *Squamanitaceae*. *Phaeolepiota* nested within *Cystoderma*; *Squamanita*, *Leucopholiota*, and *Floccularia* clustered together as two monophyletic subclades; and *Squamanita* was present as a monophyletic clade with strong statistical support in both Maximum Likelihood and Bayesian analyses. The family name *Squamanitaceae* is formally emended and a detailed taxonomic treatment is presented to accommodate the five genera. Meanwhile, another concatenated (18S-ITS-nrLSU-RPB2-TEF1*-*α) dataset is used to investigate phylogenetic relationships and species delimitation in *Squamanita*. Our data indicates that “*S. umbonata*” from the Northern hemisphere forms two species complexes, one complex includes six specimens from North America, Europe, and East Asia, the other includes two specimens from Central America and North America respectively. Futhermore, species of *Squamanita* can parasitize species of *Amanita*, besides other fungal species. *Squamanita mira* parasitizes *A*. *kitamagotake* (*A.* sect. *Caesareae*), while *S. orientalis* and *S. sororcula* are parasites of species belonging to the *A*. *sepiacea* complex (*A.* sect. *Validae*). “*Squamanita umbonata*” from Italy occurs on *A. excelsa* (*A.* sect. *Validae*). Three new species of *Squamanita* from East Asia, viz. *S. mira*, *S. orientalis* and *S. sororcula* are documented with morphological, multi-gene phylogenetic, and ecological data, along with line drawings and photographs, and compared with similar species. A key for identification of the global *Squamanita* species is provided.

**Supplementary Information:**

The online version contains supplementary material available at 10.1186/s43008-021-00057-z.

## INTRODUCTION

*Squamanita* is one of the most enigmatic genera of the Agaricales (Halama 2016; Mondiet et al. 2007; Redhead et al. 1994), and the members of this genus are extremely rare and sporadic all over the world (Griffith et al. 2019; Holden 2005; Matheny and Griffith 2010). *Squamanita* was originally described from riverine forest in Switzerland. After examining the type material, Horak (1968) presented a full re-description of the microscopic characters including features not reported in the protologue. Almost all the species of *Squamanita* are biotrophic parasites on other agaric species (Halama 2016; Harmaja 1987; Henrici 2013; Matheny and Griffith 2010; Nagasawa et al. 1990; Redhead et al. 1994; Reid 1983). The basidiomes of *Squamanita* grow from other agaric species and deform the host basidiomes so that they become incorporated into an enlarged base of the stipe of the *Squamanita*. Eventually, the host is completely deformed and more or less unrecognizable (Halama 2016; Redhead et al. 1994). Parasitized host tissue has been labelled as “sclerotial bodies”, “protocarpic tubers” (Bas 1965; Singer 1986), “galls” (Redhead et al. 1994), “cecidiocarp” (Bas and Thoen 1998) or “mycocecidium” (Griffith et al. 2019; Vizzini and Girlanda 1997), and sometimes multiple basidiomes come out from a “mycocecidium” (Bas 1965; Mondiet et al. 2007).

To date, 12 species of *Squamanita* have been accepted in the current literature (*http://www.indexfungorum.org/Names/names.asp*; Fraiture et al. 2019). It is reported that these species can parasite at least seven different genera of Agaricales, viz. *Amanita* (Bas 1965; Redhead et al. 1994), *Cystoderma* (Griffith et al. 2019; Harmaja 1987; Holden 2005; Matheny and Griffith 2010; Redhead et al. 1994; Reid 1983; Singer 1986), *Galerina* (Redhead et al. 1994), *Hebeloma* (Bas and Læssøe 1999; Mondiet et al. 2007; Vesterholt 1991), *Inocybe* (Vizzini and Girlanda 1997), *Kuehneromyces* (Cervini 2008; Gulden et al. 1977), *Phaeolepiota* (Nagasawa et al. 1990; Redhead et al. 1994), and possibly also *Mycena* (Stridvall 1994).

The genus *Squamanita* was assigned on the basis of morphology to different families in the past, including *Squamanitaceae* and *Cystodermataceae*. Based on phylogenetic analysis of combined nuclear ribosomal RNA genes, Matheny and Griffith (2010) suggested that *Squamanita*, *Cystoderma*, and *Phaeolepiota* represent a monophyletic clade. In the subsequent molecular works by Matheny et al. (2015), Griffith et al. (2019) and Vizzini et al. (2019), *Squamanita* and allied genera were referred as *Squamanitaceae*. Recently, *Squamanita*, *Cystoderma*, *Phaeolepiota*, *Floccularia*, and *Leucopholiota* were classified into *Squamanitaceae* (http://www.agaric.us) (Kalichman et al. 2020), but without a formal taxonomical treatment. In addition, the host species of *Squamanita* have been identified mainly based on morphological data and ecological evidence (Bas 1965; Mondiet et al. 2007), except for a few studies (Griffith et al. 2019; Matheny and Griffith 2010; Mondiet et al. 2007), which used molecular phylogenetic techniques to identify the hosts.

In the survey of macrofungi in China, we collected three species of *Squamanita* and two collections of *Amanita* sect. *Caesareae* and one collection of *A*. sect. *Validae* (Cui et al. 2018) with similar “mycocecidia” of two *Squamanita* species in the nearby localities respectively. To validate the taxonomical, phylogenetic and ecological traits, detailed morphological and anatomical studies and molecular phylogenetic analyses are carried out. To understand the species recorded in China, additional specimens collected in other parts of the world are examined and included in the present report.

## MATERIAL AND METHODS

### Morphology, sampling, DNA extraction, PCR amplification and sequencing

Specimens studied are listed in Tables 1 and 2. For morphological study, we follow Cui et al. (2018) and the references therein. To verify the mycoparasitic features of the target species, routine samples (HKAS100826) for DNA extraction were separately taken both from the basidiome (five samples for basidiome labeled from C1 to C5) and the mycocecidium (six samples labeled from B1 to B6 as illustrated in Fig. 6). In addition, samples of other specimens were taken from different locations from their basidiomes and mycocecidia respectively, and then mixed for improving the success probability of DNA extraction in case of poor sample quality. Particularly, the volval remnant-like structure on the cap of the *Squamanita* specimen (HKAS74862A) was sampled. All Chinese collections are deposited in the Herbarium of Cryptogams of Kunming Institute of Botany, Chinese Academy of Sciences, China (HKAS).

**Table 1:** Specimens used to infer the phylogeny of *Squamanitaceae* in this study are listed with their Herbarium ID and accession numbers. Newly generated sequences are highlighted in boldface

Taxon	Specimen	Locality	ITS	LSU	TEF1-α	RPB2	RPB1	18S
								PNS1/NS41 & NS51/NS8
*Agaricus bisporus*	H97	–	genome	genome	genome	genome	–	genome
*Agaricus campestris*	LAPAG370	Spain	KM657927	KP739803	KR006636	KT951556	–	–
*Agrocybe praecox*	AFTOL-ID 728	–	AY818348	AY646101	DQ061276	DQ385876	–	AY705956
*Alnicola luteolofibrillosa*	TU110320	Estonia	JN943976	JN938776	–	–	–	JN939103
*Amanita brunnescens*	AFTOL-ID 673	–	AY789079	AY631902	AY881021	AY780936	–	AY707096
*Auritella foveata*	TENN: 063905	India	NR_119762	GU062739	MK426177	GU062738	–	MK429932
*Bolbitius vitellinus*	AFTOL-ID 730	–	DQ200920	AY691807	DQ408148	–	–	AY705955
*Cercopemyces crocodilinus*	UTC258260	USA	JX409899	JX409897	–	–	–	–
*Chlorophyllum agaricoides*	AFTOL-ID 440	–	DQ200928	AY700187	DQ457631	–	–	AY657010
*Chlorophyllum molybdites*	Z.W.Ge 3381	USA	MG741993	MG742034	MG742091	MG742063	–	–
*Chromocyphella muscicola*	ARAN-Fungi 3210	Spain	MF623836	MF623835	MF948156	MF623838	–	–
*Conocybe lactea*	AFTOL-ID 1675	–	DQ486693	DQ457660	–	–	–	DQ437683
*Conocybe tenera*	NL-1615	–	JX968180	JX968296	JX968404	–	–	–
*Coprinellus micaceus*	FP101781	–	genome	genome	genome	genome	–	genome
*Coprinus comatus*	AFTOL-ID 626	–	AY854066	AY635772	AY881026	AY780934	–	AY665772
*Cortinarius sodagnitus*	AFTOL-ID 811	–	DQ083812	AY684151	DQ061275	DQ083920	–	AY752975
*Cortinarius violaceus*	AFTOL-ID 814	–	DQ486695	DQ457662	–	DQ470835	–	AY705950
*Crepidotus* cf. *applanatus*	AFTOL-ID 817	–	DQ202273	AY380406	DQ028581	AY333311	–	AY705951
*Crucibulum laeve*	AFTOL-ID 1334	–	DQ486696	AF336246	–	DQ470836	–	AF026624
*Cyathus striatus*	AFTOL-ID 1333	–	DQ486697	AF336247	GU187694	DQ472711	–	AF026617
*Cystoagaricus strobilomyces*	E. Nagasawa 9740 (TMI)	Japan	AY176347	AY176348	–	–	–	–
*Cystoderma amianthinum*	HKAS106238	China	**MW258857**	**MW258909**	–	**MW289802**	**MW289812**	**MW258936 & MW258887**
*Cystoderma amianthinum*	HKAS105568	China	**MW258858**	**MW258910**	–	–	**MW289813**	**MW258937 & MW258888**
*Cystoderma amianthinum*	HKAS57757	China	**MW258859**	**MW258911**	**MW324503**	**MW289803**	**MW289814**	**MW258938 & MW258889**
*Cystoderma amianthinum*	HKAS107328	China	**MW258860**	**MW258912**	**MW324504**	**MW289805**	**MW289815**	**MW258939 & MW258890**
*Cystoderma amianthinum*	HKAS107326	China	**MW258861**	**MW258913**	–	**MW289804**	**MW289816**	**MW258940 & MW258891**
*Cystoderma amianthinum*	HKAS107327	China	**MW258862**	**MW258914**	**MW324496**	**MW289806**	**MW289817**	**MW258941 & MW258892**
*Cystoderma amianthinum*	TENN: 063549	UK	GU296098	EF535265	–	–	–	GU296097
*Cystoderma amianthinum*	AFTOL-ID 1553	–	DQ192177	DQ154108	–	–	–	DQ440632
*Cystoderma* sp.	HKAS107329	China	**MW258863**	**MW258915**	**MW324497**	–	**MW289818**	**MW258942 & MW258893**
*Cystoderma superbum*	BR22288–75	Belgium	AM946504	AM946442	–	–	–	–
*Cystoderma superbum*	REG (Oct 1976)	Germany	AM946503	AM946443	–	–	–	–
*Cystodermella cinnabarina*	TAA147423	Estonia	AM946512	AM946429	–	–	–	–
*Cystodermella granulosa*	TAA147491	Estonia	AM946518	AM946431	–	–	–	–
*Descolea tenuipes*	TENN:063871	Australia	HQ832453	HQ832466	–	HQ832443	–	HQ832432
*Descomyces* sp. PDD 105133	PDD 105133	New Zealand	KP191860	KP191723	MH594070	–	–	–
*Echinoderma asperum*	HKAS 106783	North Macedonia	MN810133	MN810088	MN820902	MN820967	–	–
*Flammula alnicola*	AFTOL-ID 1501	–	DQ486703	DQ457666	GU187699	DQ472714	–	DQ113916
*Flammulaster* sp. PBM 1871	PBM 1871	–	–	AY380408	–	AY333315	–	–
*Flammulaster* sp. PBM4140	PBM4140	USA	MG773817	MT237465	–	–	–	–
*Floccularia luteovirens*	Y1	China	genome	genome	genome	genome	–	genome
*Floccularia luteovirens*	FLZJUC10	China	genome	genome	genome	genome	–	genome
*Floccularia albolanaripes*	HKAS107739	China	**MW258875**	**MW258923**	**MW324498**	**MW289809**	–	**MW258944 & MW258896**
*Floccularia albolanaripes*	HKAS107740	China	**MW258876**	**MW258924**	**MW324499**	**MW289810**	–	**MW258945 & MW258897**
*Floccularia albolanaripes*	HKAS107741	China	**MW258877**	**MW258925**	**MW324500**	**MW289811**	–	**MW258946 & MW258898**
*Galerina marginata*	AFTOL-ID 465	–	DQ192182	DQ457669	–	–	–	DQ440635
*Galerina semilanceata*	AFTOL-ID 1497	–	DQ486706	AY038309	–	AY337357	–	DQ440639
*Hebeloma mesophaeum*	KRAM:F57431	Romania	KT071038	–	KT071100	KT071077	–	–
*Hebeloma velutipes*	AFTOL-ID 980	–	AY818351	AY745703	GU187707	DQ472718	–	AY752972
*Heinemannomyces splendidissimus*	E.C. Vellinga ecv3586 (UC)	Thailand	HM488760	HM488769	–	HM488793	–	–
*Hypholoma fasciculare*	AFTOL-ID 597	–	AY818349	AY380409	–	AY337413	–	AY787215
*Inocybe myriadophylla*	AFTOL-ID 482	–	DQ221106	AY700196	DQ435791	AY803751	–	AY657016
*Inocybe rimosoides*	AFTOL-ID 520	–	DQ404391	AY702014	DQ435790	DQ385884	–	AY752967
*Inosperma calamistrata*	PBM1105	USA	JQ801386	JQ815409	MK426203	JQ846466	–	MK429958
*Kuehneromyces rostratus*	AFTOL-ID 1676	–	DQ490638	DQ457684	GU187712	DQ472730	–	DQ457624
*Laccaria laccata*	GMM7605	–	JX504146	KU685901	KU686154	KU686048	–	–
*Laccaria ochropurpurea*	AFTOL-ID 477	–	–	AY700200	–	DQ472731	–	AY654886
*Lacrymaria velutina*	AFTOL-ID 478	–	DQ490639	AY700198	–	DQ472733	–	AY654885
*Lepiota clypeolaria*	HKAS87248	China	MN810123	MN810080	MN820932	MN820941	–	–
*Leucoagaricus rubrotinctus*	HKAS54317	China	JN944082	JN940294	–	JN993685	–	JN940434
*Leucocoprinus cepistipes*	xml2014128	China	LT716023	KY418838	KY419045	KY418990	–	–
*Leucocoprinus fragilissimus*	ZRL20151466	China	LT716029	KY418844	KY419049	KY418994	–	KY418913
*Leucopholiota* aff. *decorosa*	AJ790	USA	–	MK278297	–	–	–	–
*Leucopholiota decorosa*	TENN:068830	USA	KY777364	MF797662	–	–	–	–
*Lycoperdon ericaeum*	ZRL20151498	China	LT716030	KY418845	–	KY418995	–	KY418914
*Lycoperdon perlatum*	KA13–0555	China	KP340193	–	KU764403	KU764393	–	–
*Lycoperdon pyriforme*	AFTOL-ID 480	–	AY854075	–	AY883426	AY218495	–	–
*Macrolepiota dolichaula*	AFTOL-ID 481	China	DQ221111		DQ435785	DQ385886	–	AY771602
*Macrolepiota procera*	HKAS8108	China	–	JN940277	–	JN993697	–	JN940449
*Mallocybe terrigena*	JV 16431 (WTU)	–	–	AY380401	–	AY333309	–	–
*Micropsalliota globocystis*	ZRL2013465	–	LT716024	KY418839	KY419046	KY418991	–	–
*Mycocalia denudata*	AFTOL-ID 2018	Canada	DQ911596	DQ911597	–	–	–	DQ911598
*Mythicomyces corneipes*	AFTOL-ID 972	–	DQ404393	AY745707	DQ029197	DQ408110	–	DQ092917
*Nematoloma longisporum*	AFTOL-ID 1893	–	DQ490634	DQ457681	–	–	–	DQ444863
*Nidula niveotomentosa*	AFTOL-ID 1945	Canada	DQ917654	DQ986295	–	–	–	GU296099
*Nidula* sp.	ZRL20151405	China	LT716028	KY418843	–	–	–	KY418912
*Nidularia farcta*	AFTOL-ID 1933	Sweden	GU296100	EF535276	–	–	–	–
*Nidularia farcta*	ZRL2015047	–	LT716025	KY418840	KY419047	–	–	KY418909
*Nothocybe distincta*	ZT 9250	India	KX171343	EU604546	MK426212	EU600904	–	MK429965
*Parasola conopila*	ZRL20151990	China	LT716064	KY418880	–	KY419025	–	KY418946
*Parasola conopilea*	TUB 011587	–	–	DQ071706	–	–	–	–
*Parasola plicatilis*	SZMC-NL-0295	–	FM163216	FM160693	FM897242	–	–	–
*Phaeocollybia festiva*	AFTOL-ID 1489	–	DQ494682	AY509119	–	AY509118	–	DQ462516
*Phaeolepiota aurea*	HKAS93945	China	**MW258864**	**MW258916**	**MW324501**	**MW289807**	–	**MW258943 & MW258894**
*Phaeolepiota aurea*	HKAS107738	China	**MW258865**	**MW258917**	**MW324502**	**MW289808**	–	- **& MW258895**
*Phaeomarasmius proximans*	AFTOL-ID 979	–	DQ404381	AY380410	DQ028592	AY333314	–	AY752970
*Phaeonematoloma myosotis*	SJ97002	Sweden	AF195599	AY586697	–	–	–	–
*Pholiota lenta*	PBM4233	USA	MN209743	MN251131	–	MN329707	–	–
*Pholiota squarrosa*	AFTOL-ID 1627	–	DQ494683	DQ470818	–	–	–	DQ465337
*Pholiotina filaris*	AFTOL-ID 1498	–	DQ494684	DQ470819	–	–	–	DQ465338
*Psathyloma leucocarpum*	PBM3116	New Zealand	HQ840659	HQ840660	–	HQ840662	–	HQ840661
*Psathyrella candolleana*	ZRL20151400	China	LT716063	KY418879	KY419075	KY419024	–	KY418945
*Psathyrella panaeoloides*	SZMC-NL-2537	–	FM878022	FM876279	–	–	–	–
*Psathyrella spadicea*	AFTOL-ID 1628	–	DQ494690	DQ470822	–	–	–	DQ465340
*Psathyrella spadicea*	SZMC-NL-3996	–	FN396132	FN396180	FN396231	–	–	–
*Pseudolepiota zangmui*	Z.W.Ge 2175	–	KY768928	MG742049	MG742106	KY768929	–	–
*Pseudosperma rimosum*	PBM3901	USA	JQ408772	MH220278	MK426218	MH249810	–	MK429971
*Psilocybe montana*	AFTOL-ID 820	–	DQ494692	DQ470823	–	–	–	DQ465342
*Psilocybe subaeruginosa*	PBM3218	Australia	–	KF830079	–	KF830062	–	KF830071
*Ripartitella brasiliensis*	A.E. Franco-Molano 499 (NY)	Colombia	AM946524	AM946465	–	–	–	–
*Simocybe serrulata*	AFTOL-ID 970	–	DQ494696	AY745706	GU187755	DQ484053	–	DQ465343
*Squamanita fimbriata*	LUG 12901	Switzerland	MF444998	–	–	–	–	–
***Squamanita mira***	HKAS107309A	Jiangxi, China	**MW258848**	**MW258900**	**MW324490**	**MW289797**	–	**MW258927 & MW258879**
***Squamanita mira***	HKAS107737A	Yunnan, China	**MW258849**	**MW258901**	**MW324491**	**MW289798**	–	**MW258928 & MW258880**
***Squamanita mira*** **(holotype)**	HKAS100826A	Yunnan, China	**MW258847**	**MW258899**	**MW324489**	**MW289796**	–	**MW258926 & MW258878**
*Squamanita odorata*	O-F-310485	Norway	MG711653	–	–	–	–	–
*Squamanita odorata*	O-F-146743	Norway	MG711655	–	–	–	–	–
*Squamanita odorata*	WRSL EF-2009-0001	Poland	MF444999	–	–	–	–	–
*Squamanita odorata*	K(M)178,855	England, UK	MK192934	–	–	–	–	–
*Squamanita odorata*	–	Ruaudin, France	EF091828	–	–	–	–	–
*Squamanita odorata*	DAOM225481	Honshu, Japan	–	–	–	–	–	–
***Squamanita orientalis*** **(holotype)**	HKAS74862A	Yunnan, China	**MW258851**	**MW258903**	**MW324509**	**MW289799**	–	**MW258930 & MW258881**
*Squamanita paradoxa*	GG_BM05B	Wales, UK	–	EF535266	–	–	–	–
*Squamanita paradoxa*	A. Leclerque s.n.	Belgium	MK377323	–	–	–	–	–
*Squamanita paradoxa*	herb. A. Leclerque s.n. (BR)	Belgium	MK408620	–	–	–	–	–
*Squamanita paradoxa*	TENN: 063549	Wales, UK	GU296096	–	–	–	–	GU296095
*Squamanita pearsonii*	E:204926	Scotland, UK	MK192940	–	–	–	–	–
*Squamanita pearsonii*	E:282464p	Wales, UK	MK192941	–	–	–	–	–
“*Squamanita pseudofimbriata*”	WRSL RRy-2013-0001	Poland	MF444997	–	–	–	–	–
*Squamanita schreieri* (epitype)	ZT Myc 2158	Baden-Württemberg, Germany	**MW258852**	**MW258904**	**MW324510**	**MW289801**	–	**MW258931 & MW258882**
***Squamanita sororcula*** **(holotype)**	HKAS107306A	Yunnan, China	**MW258850**	**MW258902**	**MW324507**	–	–	**MW258929 &** -
*Squamanita umbonata*	TENN:57939	North Carolina, USA	EF184305	–	–	–	–	–
*Squamanita umbonata*	DAOM199323	Rhode Island, USA	–	AF261508	–	–	–	–
“*Squamanita umbonata*”	R.E.Halling7691(NY79971)	Alajuela, Costa Rica	**MW258853**	**MW258905**	**MW324506**	**MW289800**	–	**MW258932 & MW258883**
*“Squamanita umbonata*”	H.E.Bigelow17431(NY2776224)	Massachusetts, USA	**MW258854**	**MW258906**	–	–	–	**MW258933 & MW258884**
“*Squamanita umbonata*”	C.BAS3808 (NY1840398)	Massachusetts, USA	**MW258855**	**MW258907**	–	–	–	**MW258934 & MW258885**
“*Squamanita umbonata*”	HKAS107325A	Liguria, Italy	**MW258856**	**MW258908**	**MW324508**	–	–	**MW258935 & MW258886**
*Stagnicola perplexa*	ALV17086	Denmark	MK351604	MK353788	–	MK359087	–	MK353797
*Stropharia ambigua*	AFTOL-ID 726	–	AY818350	AY646102	GU187756	DQ484054	–	DQ092924
*Tubaria confragosa*	AFTOL-ID 498	–	DQ267126	AY700190	–	DQ408113	–	AY665776
*Tubariomyces* sp.	BB6018	Zambia	MK421965	EU600887	MK426220	EU600886	–	MK429974
*Tulostoma calcareum*	GB MJ6965	Sweden	NR_164015	KU519086	KU843881	–	–	–
*Verrucospora flavofusca*	AFTOL-ID 655	China	DQ241779	DQ470825	–	–	–	AY665783

**Table 2:** Specimens used to identify the mycocecidia of new species of *Squamanita* in this study are listed with their Herbarium ID and accession numbers. Newly generated sequences are highlighted in boldface

Taxon	Specimen	Locality	ITS	LSU	TEF1-α
*Amanita* aff. *excelsa*	HKAS107325B	Italy	**MW258872** **MW258873**	**MW258922**	–
“*A*. aff. *hemibapha*”	TRTC161164	Viet Nam	–	KF877244	KF877133
“*A.* aff. *hemibapha*”	TRTC161171	Viet Nam	–	KF877245	KF877134
“*A.* aff. *hemibapha*”	BPI HPUB 560	India	–	KF877234	KF877125
“*A.* aff. *javanica*”	HKAS56957	China	JX998039	JX998068	JX998017
“*A.* aff. *javanica*”	HKAS56863	China	JX998040	JX998071	JX998014
“*A.* aff. *javanica*”	HKAS53281	China	JX998041	JX998070	JX998016
*A.* aff. *sepiacea* sp. 1	HKAS107306B	China	**MW258871**	–	**MW324505**
*A.* aff. *sepiacea* sp. 2	HKAS74861	China	**MW258869**	–	–
*A.* aff. *sepiacea* sp. 2	HKAS74862B	China	**MW258870**	–	–
*A. arkansana*	RET-354-9	USA	JX844674	KF877197	KP724414
*A. brunneolimbata*	HKAS78459	China	MH508274	–	–
*A. brunneolimbata*	HKAS101392	China	MH508272	–	–
*A. brunneolimbata*	HKAS78460	China	MH508275	–	–
*A. caesarea*	RET-4271-1	Italy	JX844685	KF877207	KF877106
*A. caesaroides*	RET-356-10	China	–	KF877209	KF877107
*A. cinnamomescens* (isotype)	RET-290-5	Pakistan	JX844699	KF877221	KF877114
*A. citrina*	HKAS53467	Germany	MH508312	–	–
*A. cochiseana* nom. prov.	RET-498-1	USA	JX844705	KF877226	KP724516
*A. fritillaria*	HKAS100521	China	MH508360	–	–
*A. fritillaria*	HKAS100520	China	MH508359	–	–
*A. garabitoana* (paratype)	RET-333-6	Costa Rica	JX844711	KF877231	KF877122
*A. hemibapha*	RE-342-8	India	JX844716	KF877233	KF877124
*A. jacksonii*	RET-393-7	USA	JX844724	KF877252	KP724554
“*A. javanica*”	S-170	Japan	LC056770	LC056748	–
“*A. javanica*”	S-329	Japan	LC056772	–	LC164656
“*A. javanica*”	S-76	Japan	AB750726	LC164652	LC164654
*A. kitamagotake*	HKAS100824	China	**MW258866**	**MW258918**	**MW324492**
*A. kitamagotake*	HKAS100825	China	**MW258867**	**MW258919**	**MW324493**
*A. kitamagotake*	HKAS107309B	China	**MW258874**	**MW258921**	**MW324495**
*A. kitamagotake*	HKAS100826B	China	**MW258868**	**MW258920**	**MW324494**
*A. kitamagotake* (ex-holotype)	EN-4	Japan	AB721450	AB721450	LC164658
*A. porphyria*	HKAS92088	China	MH508506	–	–
*A. porphyria*	MB-100156	Germany	MH508507	–	–
*A. rubromarginata* (isotype)	RET-383-1	Japan	JX844739	KF877279	KF877164
*A. sepiacea*	HKAS80970	China	MH508589	–	–
*A. sepiacea*	HKAS79669	China	MH508588	–	–
*A. sepiacea*	HKAS74750	China	MH508587	–	–
*A. sepiacea*	HKAS70045	China	MH508586	–	–
*A. sepiacea*	HKAS68614	China	MH508585	–	–
*A. sepiacea*	HKAS56799	China	MH508584	–	–
*A. sepiacea*	HKAS100604	China	MH508582	–	–
*A. sinocitrina*	HKAS100530	China	MH508598	–	–
*A. sinocitrina*	HKAS83445	China	MH508601	–	–
*A. sinocitrina*	HKAS100531	China	MH508599	–	–
*A. vernicoccora* (paratype)	7020	USA	GQ250401	GQ250416	–

The total genomic DNA of all the materials of the parasitic species and the coexisting *Amanita* species was extracted by using the Extract-N-Amp kit (Sigma, USA). Universal primer pairs LROR/LR5 (Vilgalys and Hester 1990), ITS1F/ITS4 (Gardes and Bruns 1993; White et al. 1990), PNS1/NS41 (Bruns lab; Hibbett 1996) and NS51/NS8 (Bruns lab; White et al. 1990), and EF1-983F/EF1-1567R (Rehner and Buckley 2005), RPB2-6F/RPB2-7R (Hall lab), RPB1-Af/RPB1-Dr (Hall lab) were used for amplifying the large nuclear ribosomal RNA subunit (nrLSU), the internal transcribed spacers 1 and 2 with the 5.8S rDNA (ITS), the small subunit (18S) region, translation elongation factor 1-α (TEF1*-*α), the RNA polymerase II second largest subunit (RPB2), and RNA polymerase II largest subunit (RPB1) respectively.

PCR products which failed in direct sequencing were firstly purified with the Cycle-pure-kit (Omega, USA) or Gel Extraction and PCR Purification Combo Kit (Spin-column) (Bioteke, China), and then cloned using pClone007 simple vector kit (Tsingke, Beijing). For the recently collected specimen (HKAS100826) and the volval remnants like structure on the cap of a *Squamanita* specimen (HKAS74862A), 10 clones of each ITS and nrLSU PCR products of each sampling point were randomly selected from a 90 mm petri dish for sequencing with primer pair M13–47/M13–48 to investigate the mycelium distribution of hosts and parasitising fungi. The cloning, PCR amplification and sequencing followed the protocols described by Cai et al. (2016) and Cui et al. (2018).

### Results of sequencing

**Fig. 1:**
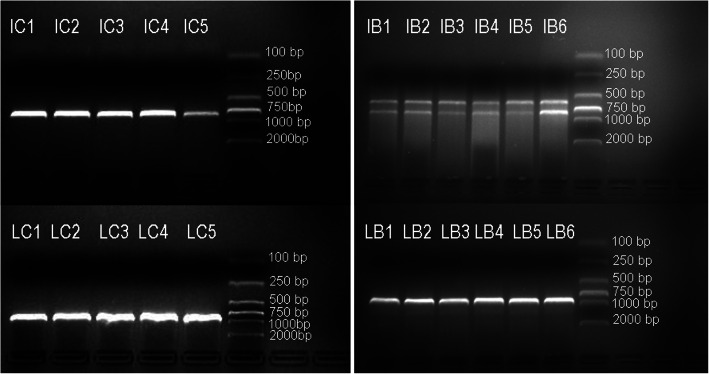
Gel electrophoresis diagram of the PCR products amplified from 11 sampling points on the basidiome (C1, C2, C3, C4, C5) and mycocecidium (B1, B2, B3, B4, B5, B6) of *Squamanita mira* (HKAS100826, holotype), as indicated on Fig. 6. I and L indicate ITS and LSU (nrLSU), respectively

**Fig. 2:**
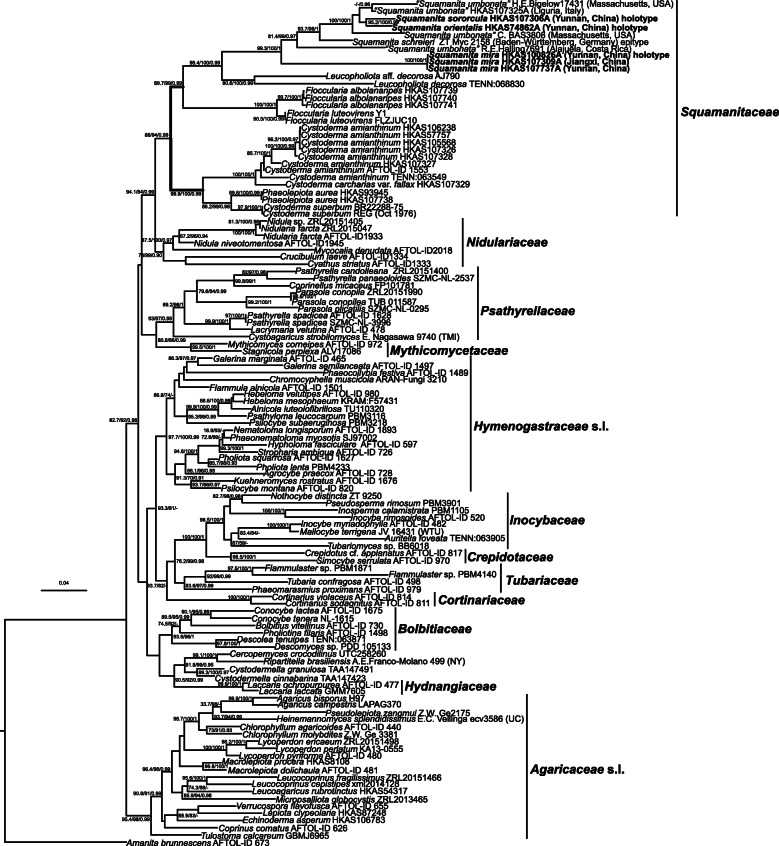
Maximum-Likelihood (ML) phylogenetic tree of Squamanitaceae inferred from the 18S-5.8S-nrLSU-RPB2-TEF1*-*α dataset, with SH-aLRT (left), ultrafast bootstrap (UFB) (middle), and PPs values (right) near by the corresponding node. Only one of SH-aLRT > 80 or UFB > 95 for ML and PPs > 0.90 for BI are indicated along branches (SH-aLRT/UFB/PP). New species *Squamanita mira*, *S. orientalis*, *S. sororcula* are highlighted in boldface

**Fig. 3:**
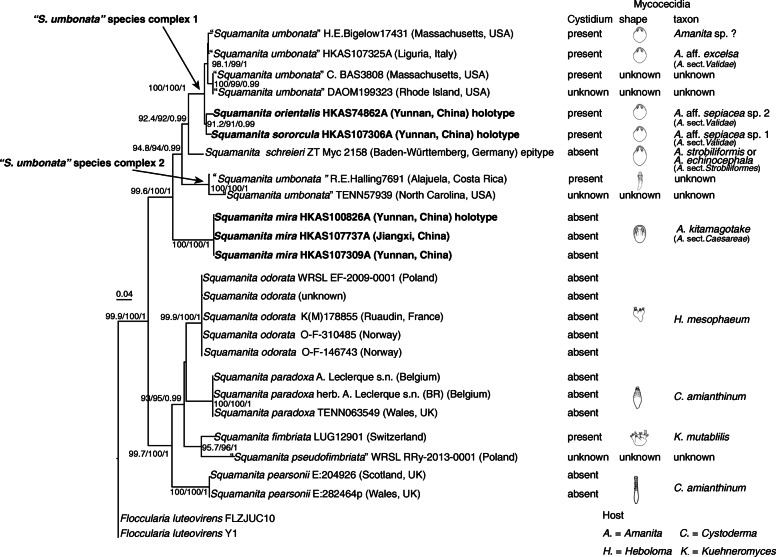
Maximum-Likelihood (ML) phylogenetic tree of *Squamanita* inferred from the 18S-ITS-nrLSU-RPB2-TEF1*-*α dataset, with SH-aLRT (left), ultrafast bootstrap (UFB) (middle), and PPs values (right) near by the corresponding node. Only one of SH-aLRT > 80 or UFB > 95 for ML and PPs > 0.90 for BI are indicated along branches (SH-aLRT/UFB/PP). Cystidium present or absent, and the shape and taxon of mycocecidia of counterpart *Squamanita* species are shown. New species *Squamanita mira*, *S. orientalis*, *S. sororcula* are highlighted in boldface

**Fig. 4:**
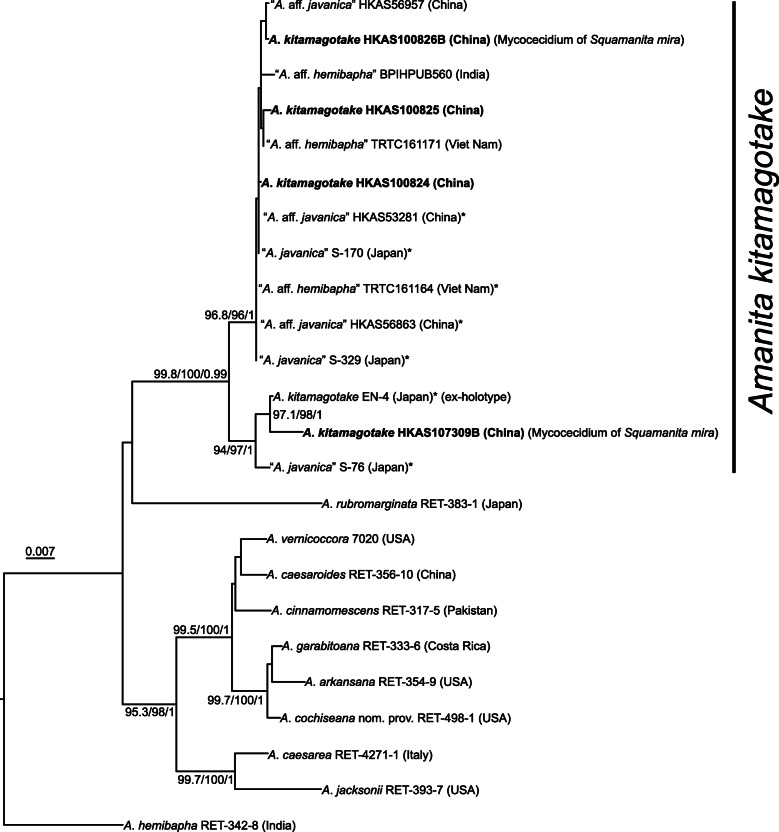
Maximum-Likelihood (ML) phylogenetic tree inferred from the ITS-nrLSU-TEF1*-*α dataset for detecting the phylogenetic relationships of the mycocecidia (hosts) of *Squamanita mira* and two specimens (HKAS100824 and HKAS100825) of *Amanita kitamagotake* collected near to *S. mira* (these four specimens are highlighted in boldface), with SH-aLRT (left), ultrafast bootstrap (UFB) (middle), and PPs values (right) near by the corresponding node. Only one of SH-aLRT > 80 or UFB > 95 for ML and PPs > 0.90 for BI are indicated along branches (SH-aLRT/UFB/PP). The sequences which were regarded as *A*. *kitamagotake* in Endo et al. (2017) are marked by asterisks (*)

**Fig. 5:**
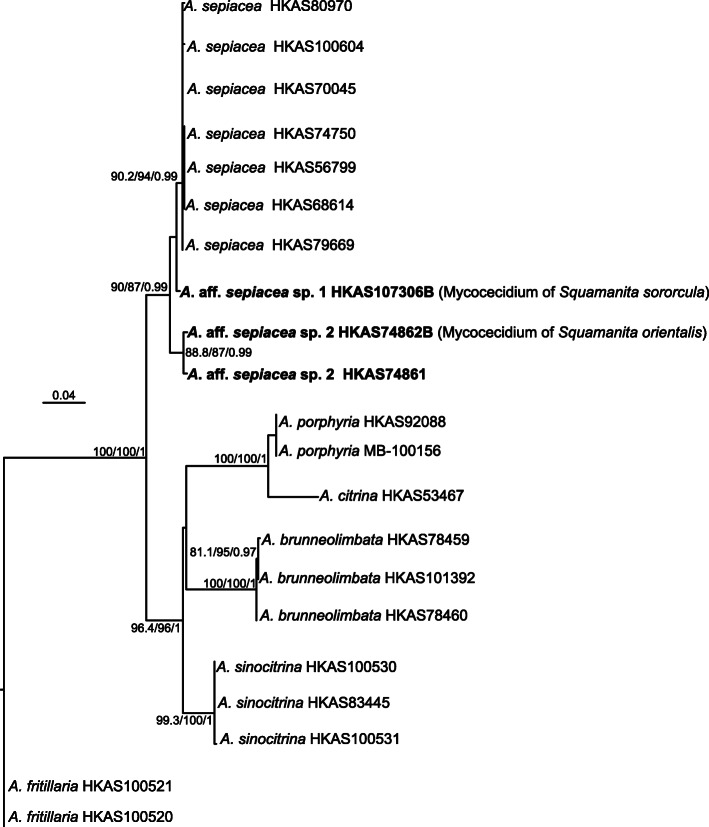
Maximum-Likelihood (ML) phylogenetic tree inferred from the ITS dataset for detecting the phylogenetic relationships of the mycocecidia (hosts) of *Squamanita orientalis*, *S. sororcula* and one specimen (HKAS74861) of *Amanita sepiacea* that was collected nearby *S. orientalis* (these three specimens are highlighted in boldface), with SH-aLRT (left), ultrafast bootstrap (UFB) (middle), and PPs values (right) near by the corresponding node. Only one of SH-aLRT > 80 or UFB > 95 for ML and PPs > 0.90 for BI are indicated along branches (SH-aLRT/UFB/PP)

For specimen of HKAS100826, the ITS and nrLSU sequences were successfully amplified from all eleven sampling points (C1–C5, B1–B6). Among them, there are two bands occurring in gel electrophoresis diagram of each of the PCR products of ITS from six sampling points of mycocecidium (B1, B2, B3, B4, B5, B6), see Fig. 1. By cloning and sequencing all of the purified PCR products of ITS and nrLSU, a total of 50 ITS and 50 nrLSU sequences were generated from all points (C1–C5). After alignment and comparison, all of them belong to the same species, namely the mycoparasitic species itself. For the mycocecidium, each band of PCR productions with two bands were excised from gel respectively, and then purified and sequenced, generating a total of 120 ITS and 60 nrLSU sequences from sampling points B1–B6. After analysis, two types of mushroom sequences were detected for each DNA locus. Statistically, 50% ITS, 90% nrLSU matched to the potential mycoparasitic species and 50% ITS, 10% nrLSU belong to the potential host species. For the volval remnants on the cap of the *Squamanita* specimen (HKAS74862A), 60% ITS, 90% nrLSU were the potential mycoparasitic species and 20% ITS, 0% nrLSU were assigned to the potential host species, others are *Trichoderma hirsutum* or vector sequences. For the other specimens of *Squamanita* and nearby *Amanita*, all sequences were amplified then directly sequenced or obtained by cloning from PCR products. One hundred forty-five sequences have been submitted to GenBank and used for phylogenetic analyses (Tables 1 and 2). The sequences of the two potential species of hosts are the same as those of the coexisting *Amanita* species respectively, and were finally identified to belong to *A*. *kitamagotake* (Fig. 4) and the *A*. *sepiacea* complex (Fig. 5). The potential mycoparasitic species are clustered into the genus *Squamanita* (Figs. 2 and 3).

### DNA sequence alignment

Sequences used in study are listed in Tables 1 and 2 with their Herbarium ID and accession numbers. Four datasets, namely 18S-5.8S-nrLSU-RPB2-TEF1*-*α, 18S-ITS-nrLSU-RPB2-TEF1*-*α, ITS-nrLSU-TEF1*-*α, and ITS were used in our study to reinvestigate the phylogeny of *Squamanitaceae*, identify the phylogenetic position of the basidiomes and mycocecidia of the mycoparasitic species. From the first dataset to the last, a total of 4100, 4743, 1878 and 693 characters were used in the phylogenetic analyses, respectively. Moreover, two phylogenetic trees which only use ITS and nrLSU sequences were used to investigate the phylogeny of *Squamanitaceae* are provided as additional files (Additional files 1 and 2), respectively. The final alignments have been submitted to TreeBase (https://www.treebase.org/, nos.: 27,493, 27,494, 27,496, 27,497, 27,498, 27,499).

For each dataset, the sequences were aligned using MAFFT v6.8 (Katoh et al. 2005), manually edited with BioEdit v7.0.9 (Hall 1999) and concatenated with Phyutility v2.2.1 (Smith and Dunn 2008). Unsampled gene regions were coded as missing data. In the concatenated datasets, all introns of RPB2 and TEF1-α were excluded because of the difficulty in alignment. Maximum likelihood (ML) analyses were performed using IQ-TREE 1.6 (Trifinopoulos et al. 2016). Bayesian Inference (BI) analyses were used to analyze the datasets with MrBayes v3.1.6 (Ronquist et al. 2012). The optimal substitution models for each dataset were determined by using the Akaike Information Criterion (AIC) implemented in MrModeltest v2.4 (Nylander 2004), with 18S, 5.8S/ITS and nrLSU treated as a single block. In ML analyses, the substitution model options for four datasets were auto evaluated after provided partition file by using IQ-TREE 1.6 (http://iqtree.cibiv.univie.ac.at/), clade support for the ML analyses was assessed using an SH-aLRT test with 1000 replicates (Guindon et al. 2010) and 1000 replicates of the ultrafast bootstrap (UFB) (Hoang et al. 2018). In the ML analyses, nodes with support values of both SH-aLRT ≥80 and UFB ≥ 95 were considered well supported, nodes with one of SH-aLRT ≥80 or UFB ≥ 95 were weakly supported, and nodes with both SH-aLRT < 80 and UFB < 95 were unsupported, and the other parameters use the default settings. For BI analyses, the selected models for four datasets were 18S–5.8S-nrLSU(GTR + I + G)-RPB2(GTR + I + G)-TEF1*-*α(GTR + I + G), 18S-ITS-nrLSU(GTR + I + G)-RPB2(SYM + I)-TEF1*-*α(SYM + I + G), ITS(SYM + G)-nrLSU(HKY + I)-TEF1*-*α(SYM + G), and ITS (GTR + G) respectively. Bayesian analyses used the selected models and four chains were run simultaneously for 2 million generations with trees sampled every 100 generations. The sampling of the posterior distribution was considered to be adequate when the average standard deviation of split frequencies was lower than 0.01. Chain convergence was determined by checking the effective sampling size (ESS > 200) in Tracer v. 1.5 (Rambaut and Drummond 2009). Nodes with Bayesian posterior probability (PP) > 0.90 were considered well supported. Subsequently, trees are summarized and posterior probabilities were obtained by using the sumt and sump command implemented in MrBayes by discarding the first 25% generations as burn-ins.

## RESULTS

For the four datasets, topologies of the phylogenetic trees generated from ML and BI analyses are nearly identical with minimal variation in statistical support values, and thus only the trees inferred from the ML analyses are displayed. The tree generated from the 18S–5.8S-nrLSU-RPB2-TEF1*-*α dataset reveals that *Squamanita*, *Cystoderma*, *Phaeolepiota*, *Floccularia*, and *Leucopholiota* form a monophyletic clade with weakly statistical support in ML analysis but with strong statistical support in BI analysis (SH-aLRT/UFB/PP = 88/94/0.99), *Squamanita* and *Leucopholiota* are sister groups of *Floccularia* (SH-aLRT/UFB/PP = 98.4/100/0.99), *Phaeolepiota* nested within *Cystoderma* (SH-aLRT/UFB/PP = 99.9/100/0.99), and *Squamanita* is a monophyletic group with strong statistic support in both of ML and BI analyses (SH-aLRT/UFB/PP = 99.3/100/1) (Fig. 2). Taking the study of Matheny and Griffith (2010) and Kalichman et al. (2020) into consideration, the family *Squamanitaceae* is formally emended to accommodate the above-mentioned five genera. Besides, both trees generated from 18S–5.8S-nrLSU-RPB2-TEF1*-*α and 18S-ITS-nrLSU-RPB2-TEF1*-*α datasets reveal that the three potential *Squamanita* species from China are novel (Figs. 2 and 3). They are described below as *S. mira*, *S. orientalis* and *S. sororcula*, respectively. The tree generated from the 18S-ITS-nrLSU-RPB2-TEF1*-*α dataset also shows that several “*S. umbonata*” from North America, Europe and East Asia harbor a complex of species, with six subclades in the phylogenetic tree (Fig. 3), and one “*S. umbonata*” from Central America harbors a monophyletic clade with a sequence from North Carolina, USA (Fig. 3). The trees generated from ITS-nrLSU-TEF1*-*α and ITS datasets reveal that the host of *S. mira* is *A*. *kitamagotake* (Fig. 4), and those of *S. orientalis* and *S. sororcula* are species of the *A*. *sepiacea* complex (Fig. 5).

## TAXONOMY

***Squamanitaceae*** Jülich, *Biblthca Mycol.* **85**: 390 (1981).

*Type*: *Squamanita* Imbach, *Mitt. Naturf. Ges. Luzern* **15**: 81 (1946).

*Synonym*: *Cystodermataceae* Locq., *Mycol. gén. struct*.: 108 (1984); nom. inval. (Art. 36.1, lacking a Latin diagnosis or reference to a previously published Latin diagnosis).

*Emended description: Basidiome* lepiotoid to tricholomatoid, small to medium-sized, with pileus and central stipe; lamellae adnexed to adnate, or with decurrent tooth, never free. *Stipe* with or without annulus. *Mycocecidia* subglobose or subcylindrical to clavate fusiform. Stipe and pileus often with a floccose layer composed of loose sphaerocysts. *Hyphal system* monomitic. *Hyphae* cylindrical or slightly inflated, thin-walled, smooth, with clamps. *Cystidia* absent or present; if present, thin- to slightly thick-walled, smooth. *Basidia* narrowly clavate, 4-spored. *Basidiospores* subglobose to ellipsoid or subreniform, rarely angular, thin- to slightly thick-walled, colorless, yellowish or brownish mostly smooth, in some taxa finely verrucose to finely echinulate, without germ pore, amyloid or inamyloid, not or slightly dextrinoid. *Conidia* present or absent, if present, globose, subglobose, ovoid, irregularly clavate, cylindrical, ellipsoid, broadly fusiform or fusiform, 7–16 (− 19) × 4–7.5 (− 12.5) μm, with clamps when young, later more or less bifid at base, colourless to pale brownish yellow, smooth or ornamented, with thickened wall, development of conidia basifugal. *Conidiophores* colourless, septate when young, thin walled, densely branching, 4–6 μm wide, with clamps, the older conidia-bearing branches non-septate, sickle-shaped. *Chlamydospores* present or absent, if present, obovoid, clavate, rarely ventricose-fusiform, rectangular to variously shaped, inamyloid, colorless to yellowish, thick-walled.

*Substrate*: On soil, wood or parasitizing agarics.

*Genera included*: *Squamanita*, *Cystoderma*, *Phaeolepiota*, *Leucopholiota*, and *Floccularia*.

*Notes*: Here we fix the application of the generic name *Squamanita* by lecto- and epitypfiying the type species of the genus, *S. schreieri*, and describe the new species discovered in this study.

***Squamanita schreieri*** Imbach, *Mitt. Naturf. Ges. Luzern* **15**: 81 (1946).

*Type*: Imbach, *Mitt. Naturf. Ges. Luzern* **15**: 80 [un-numbered plate] (1946) –lectotype designated here (MBT 394854). Germany: Baden-Württemberg, Taubergiessen Nature Reserve, Alluvial forest, close to a *Populus* tree, 10 Oct 1991, Leg. M. Wilhelm (no. 295) *ZT Myc 2158* – epitype designated here (MBT 394983).

*Notes*: The original description cited the following collections: “Schreier, 17 Jul. 1935; Schreier, 4 Aug. 1936; Schreier, 8 Aug. 1937; Arndt, 11 Jul. 1942; Haller, 17 Oct. 1943; Furrer, Schlapfer & Imbach, 18 Jul. 1944; Rohl-Wütherich, Aarau & Imbach, 31 Jul. 1945”. None of these original collections could be located in G, and the only remaining original material is the illustration provided by Imbach which is therefore designated as lectotype here. As a specimen is essential to fix the application of the name, we designate as an epitype a modern collection in Eidgenössische Technische Hochschule Zürich which fits the original diagnosis and plate.

**Fig. 6:**
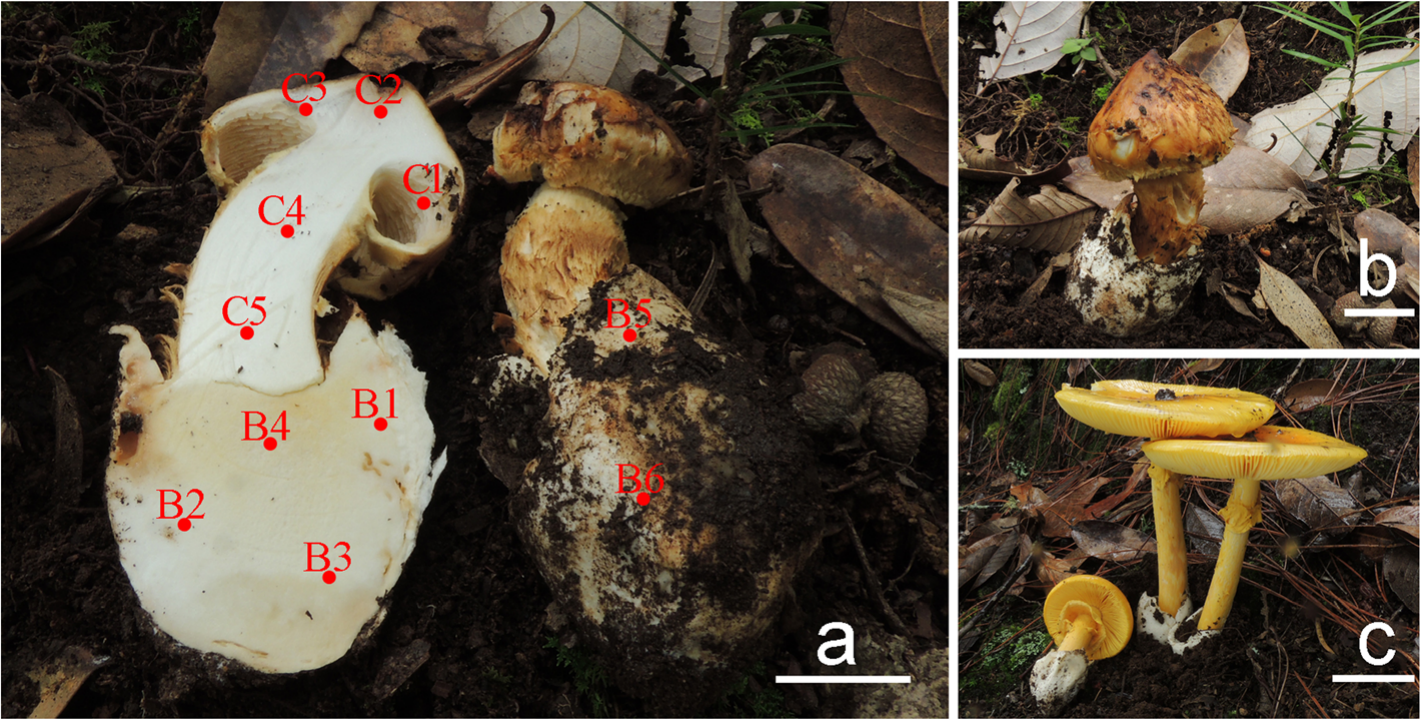
**a**–**b** Basidiomes of *Squamanita mira* HKAS100826 (holotype) photos by Jian-Wei Liu. Bars: 20 mm. **c** Basidiomes of *Amanita kitamagotake*. HKAS100825. Photos by Jian-Wei Liu. Bars: 50 mm. Sampling points are marked by red dots labelled C1, C2, C3, C4, C5, (from basidiome) and B1, B2, B3, B4, B5, B6 (from mycocecidium)

**Fig. 7:**
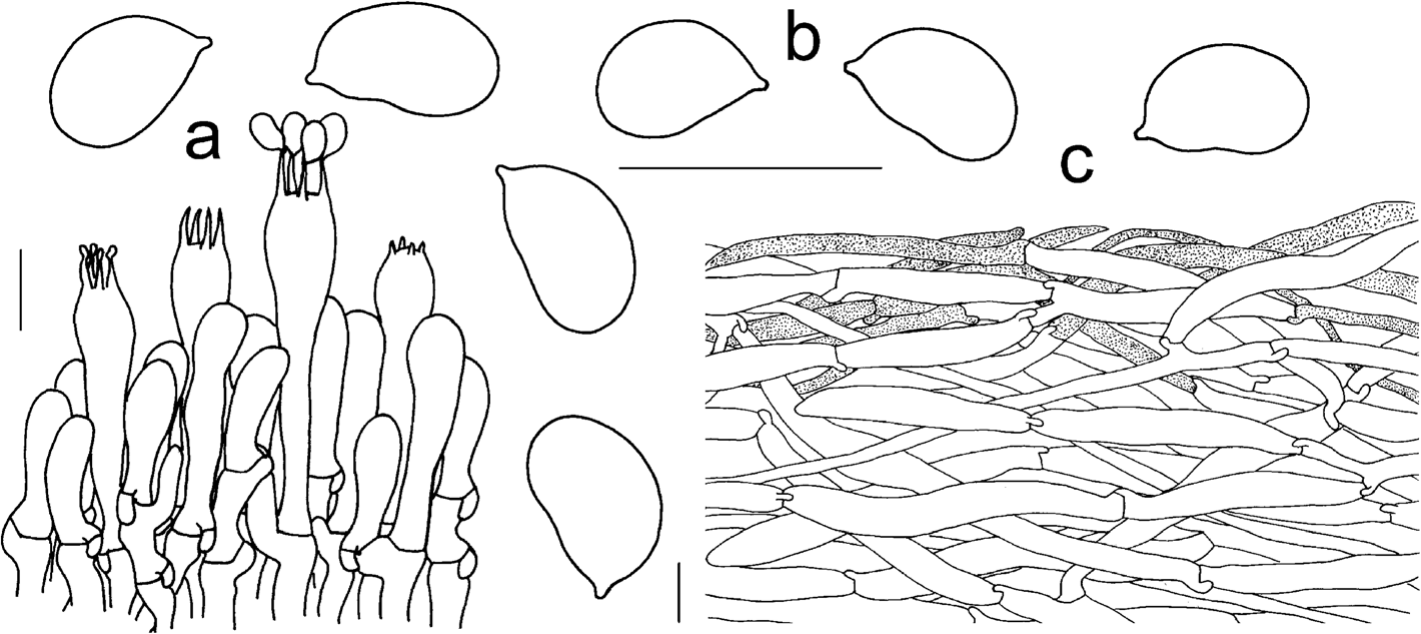
Microscopic features of *Squamanita mira* (HKAS100826A, holotype). **a** Hymenium and subhymenium. **b** Basidiospores. **c** Pileipellis section. Bars = 10 μm. Drawings by Jian-Wei Liu

***Squamanita mira*** J. W. Liu & Zhu L. Yang, ***sp. nov.*** — *Fungal Names* FN570781;

MycoBank 836,584. (Figs. 6 and 7).

*Etymology*: —*mirus* (Lat.), wonderful or extraordinary, referring to the wonderful basidiome.

*Diagnosis*: *S. mira* differs from other species of the genus by the mycocecidia which have a limbate volva-like structure and the absence of cystidia.

*Type*: **China**: *Yunnan Province*: Ailaoshan Natural Reserve, Chuxiong, Nanhua, in the forests dominated by *Fagaceae* and *Pinaceae*, 24°54′27.53″N, 100°49′14.91″E, 2235 m elev., 10 Aug. 2017, *J. W. Liu 904* (HKAS100826A – holotype; GenBank Acc. nos.: 18S = MW258926 & MW258878, ITS = MW258847, nrLSU = MW258899, TEF1-α = MW324489, RPB2 = MW289796).

*Description*: *Pileus* ca. 40 mm diam, subconical to convex, distinctly umbonate; surface dry, yellowish brown (6C6–7) or honey-yellow (6C6–8), or viscid if moist, covered with dark orange (6A8), yellow-tawny (6B7–8) or honey yellow (6C6–8), repent, fibrillose squamules; margin incurved, strongly appendiculate, irregularly and densely corniform and fibriform squamules derived from breaking up of the veil, and slightly paler than the pileus surface. *Lamellae* adnexed to adnate, moderately crowded, narrow; edge irregularly serrate-dentate or subundulate. *Stipe* 43–46 *×* 12–24 mm, subcylindrical, densely covered with brown (6A7–8), tawny yellow (6B7–8) to yellowish brown (5A6–8), appressed or recurved fibrillose and villiform squamules, at the upper part of the stipe covered with fluffy and villose, brown (5A6–8), tawny yellow (6B7–8) to yellowish brown (6C6–7) appressed or erect, fibrillose or obliquely lacerate scales arranged in irregular rings, 4–6 mm from apex, extreme apex off-white (1A1–2) and subglabrous. *Mycocecidia* subglobose to napiform, 40–46 *×* 5–16 mm, nearly smooth, whitish (1A1) or locally yellow (6A4–5) on external surface; *Volval limb* arising from margin of mycocecidia, 6–20 mm tall; context of pileus and stipe white (1A1), with a strong aromatic smell, like that of *Tricholoma matsutake*; context of mycocecidia white, unchanging on exposure, odour not distinctive.

*Basidiospores* [60/1/1] (5.5–) 6–7 (− 7.5) × 4–5 (6) μm, (Q = (1.16) 1.33–1.75 (− 1.8), Qm = 1.53 ± 0.13), ellipsoid or subreniform, colorless, hyaline, smooth, inamyloid. *Basidia* 22–65 × 9–12 μm, fusiform to ventricose-fusiform, hyaline; sterigmata 4–5 μm long; *Cystidia* absent. *Subhymenium* 10–20 μm thick, composed of 4–7 μm wide filamentous hyphal segments. *Lamellar trama* regular, composed of colorless, thin-walled hyphae 4–17 μm diam, branching, sometimes anastomosing. *Pileipellis* a cutis with transition to a trichoderm at regular intervals, composed of loosely and more or less radially arranged, thin-walled hyphae 90–200 (− 370) × 5–20 μm, and upper part of pileipellis often with fine brownish granular incrustations and yellowish to brownish filamentous hyphae, constricted at septa; *Mycocecidia* composed of abundant ovoid to subglobose inflated cells, and filamentous hyphae similar to those on the pileus, clamp connections present; chlamydospores not observed.

*Ecology*: Parasitic on *Amanita kitamagotake* (HKAS100826B, GenBank Acc. nos.: ITS = MW258868, nrLSU = MW258920, TEF1-α *=* MW324494; HKAS107309B, GenBank Acc. nos.: ITS = MW258874, nrLSU = MW258921, TEF1-α *=* MW324495) growing on soil under trees of *Fagaceae* and *Pinaceae*.

*Distribution*: Currently known from Jiangxi and Yunnan Province, central and Southwest China.

*Notes*: In this study, molecular evidence confirms that the hosts of *S. mira* as well as two collections of *Amanita* in the nearby area, within 2 km of *S. mira,* are *A*. *kitamagotake* (Figs. 4, 6).

Morphologically, *S. mira* highly resembles the informally published “*S. tropica*” (“nom. Prov.”) (Bas 1965), because both are parasitic on basidiomes of *Amanita* and form a volva-like structure at the base of the stipe. Furthermore, they share abundant tawny squamules on the pileus surface, serrate-dentate or subundulate lamellae edges, irregular ring analogues on the upper part of the stipe and ellipsoid to subreniform basidiospores. However, *S. mira* differs from *S. tropica* in its subconical to convex pileus with a distinct umbo. The material of *S. tropica* is lost (Bas 1965).

*Squamanita mira* is also more or less similar to *S. schreieri* and the specimens under the two species complexes of “*S. umbonata*” from all over the world in some morphological features*.* However, *S. mira* can be distinguished from the aforementioned taxa by its mycocecidia with a limbate volva-like structure and absence of cystidia. Phylogenetically, they are grouped, however, in different clades (Figs. 2 and 3).

*Additional specimens examined*: **China**: *Jiangxi Province*: Jian, Jinggangshan City, Jinggangshan scenic spots, 800–900 m elev. 19 July. 2019, *Chunlei Pan JGS001* (HKAS107309A, GenBank Acc. nos.: 18S = MW258927 & MW258879, ITS = MW258848, nrLSU = MW258900, TEF1-α *=* MW324490*,* RPB2 = MW289797). *Yunnan Province*: Ailaoshan Natural Reserve, Chuxiong, Nanhua, in the forests dominated by *Fagaceae* and *Pinaceae*, 24°53′46.23″N, 100°48′11.14″E, 2339 m elev., 11 Aug. 2020, *LCC002* (HKAS107737A, GenBank Acc. nos.: 18S = MW258928 & MW258880, ITS = MW258849, nrLSU = MW258901, TEF1-α *=* MW324491*,* RPB2 = MW289798).

**Fig. 8:**
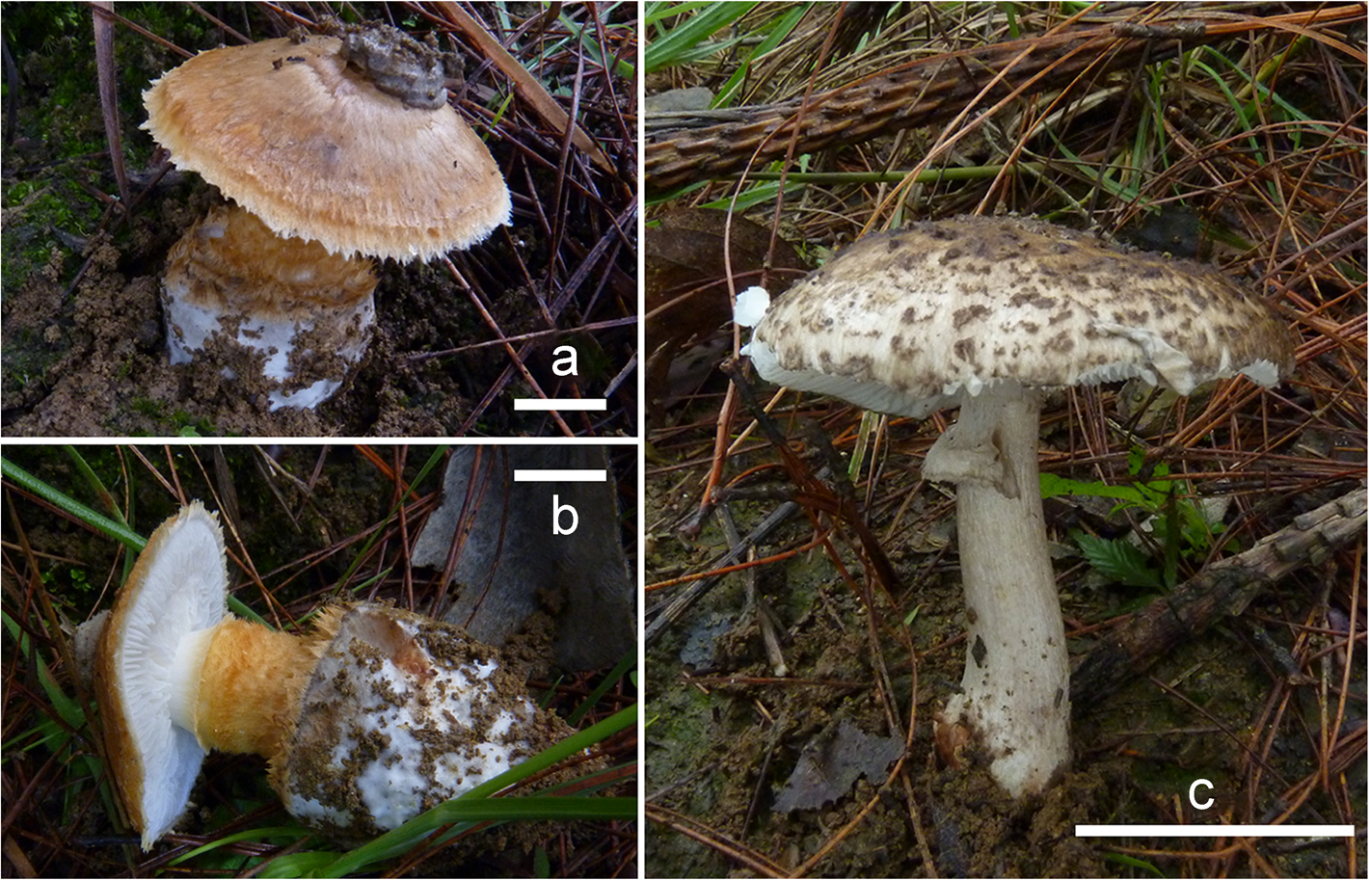
**a**–**b** Basidiomes of *Squamanita orientalis* (HKAS74862A, holotype). Photos by Gang Wu. Bars: 10 mm. A lump of clay is attached on the center of pileus, and the apical part of volval remnants on mycocecidiium can be observed between clay and pileus under anatomical lens. **c** Basidiome of *Amanita sepiacea* (HKAS74861). Photos by Gang Wu. Bars: 50 mm

**Fig. 9:**
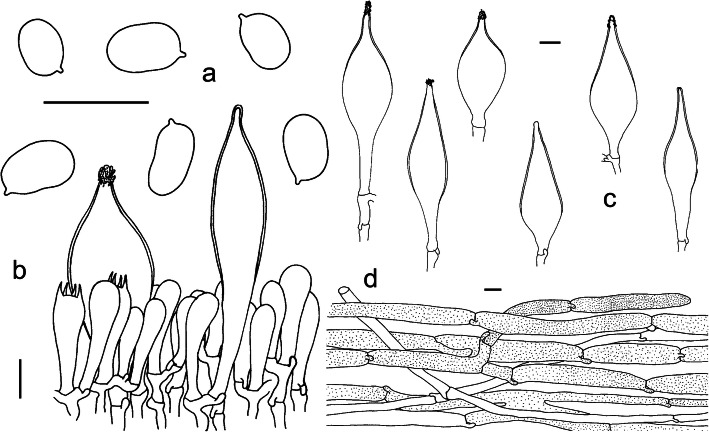
Microscopic features of *Squamanita orientalis* (HKAS74862A, holotype). **a** Basidiospores. **b** Hymenium and subhymenium with one of the two pleurocystidia covered with refractive incrustations. **c** Pleurocystidia, four of them covered with refractive incrustations. **d** Pileipellis section. Drawings by Jianwei Liu. Bars = 10 μm

**Fig. 10:**
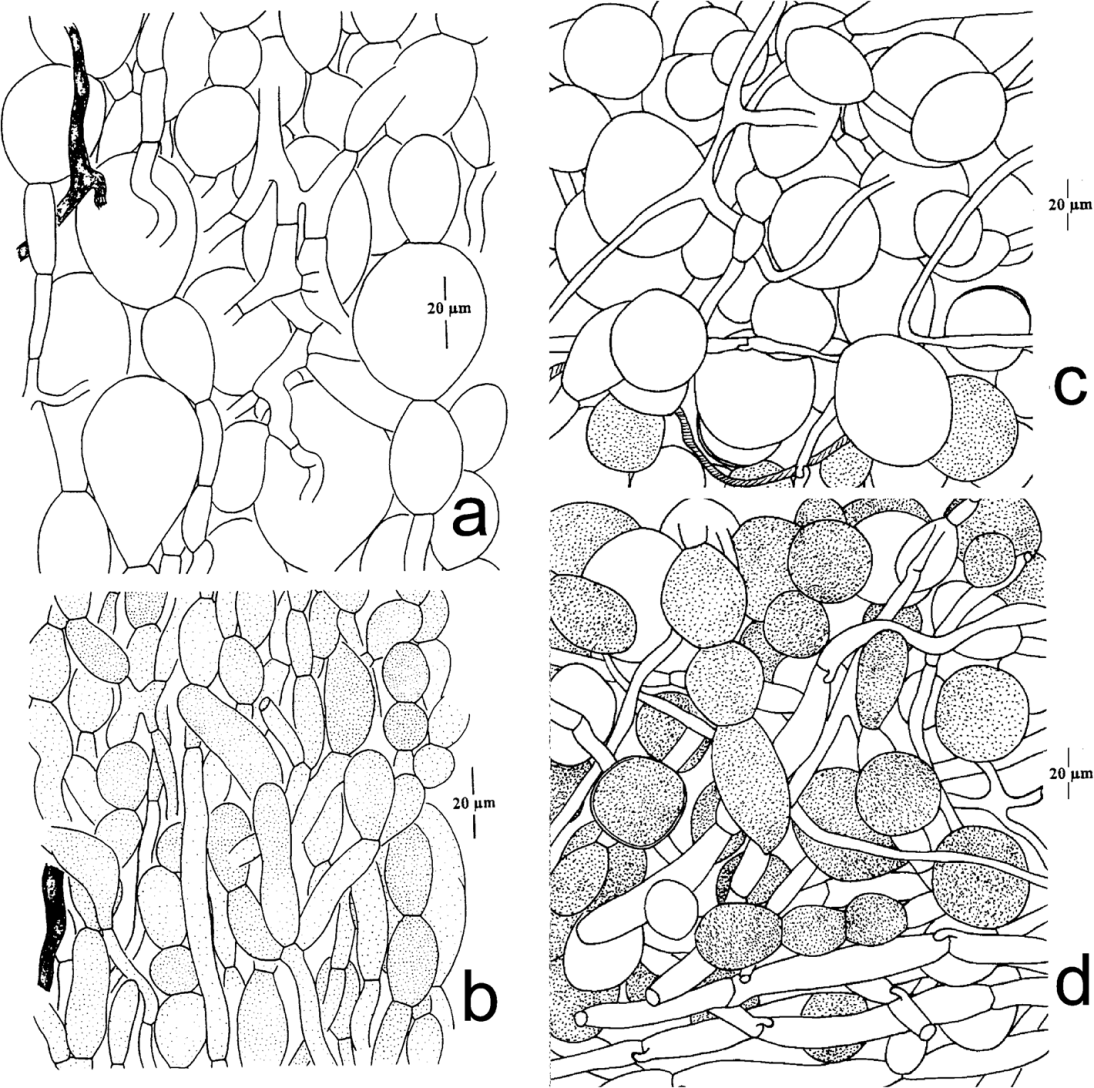
Microscopic features of volval remnants on the pileus of *Amanita sepiacea* (HKAS32519) and *Squamanita orientalis* (HKAS74862A, holotype). **a** the upper part of a volval remnant on the pileus of *A*. *sepiacea*. **b** the lower part of a volval remnant on the pileus of *A*. *sepiacea*. Drawings by Zhuliang Yang (2005). **c** Upper layer of a volval remnant on the pileus of *S. orientalis*. **d** Inner layer of a volval remnant on the pileus of *S. orientalis*. Drawings by Jianwei Liu. Bars = 20 μm. Cells with vacuolar pigment and vascular hyphae are dotted and shaded respectively. Hyphae with clamps belong to *S. orientalis*

***Squamanita orientalis*** J. W. Liu & Zhu L. Yang, ***sp. nov.*** — *Fungal Names* FN570782;

MycoBank 836585. (Figs. 8, 9 and 10).

*Etymology*: —*orientalis* (Lat.): from the East.

*Diagnosis*: *S. orientalis* differs from other species by its irregular fibrillose annular zone on the upper part of the stipe and ciliate squamules on the pileal margin, larger cystidia (90–105 × 17–27 μm), and subglobose mycocecidia.

*Type*: **China**: *Yunnan Province*: Laowopo dunk, Chongren, Nujiang, 1700–1800 m elev., in forest dominated by *Fagaceae* and *Rhododendron*, 7 Aug. 2011, *Gang Wu 548* (HKAS74862A – holotype; GenBank Acc. nos.: 18S = MW258930 & MW258881, ITS = MW258851, nrLSU = MW258903, TEF1-α *=* MW324509*,* RPB2 = MW289799).

*Description*: *Pileus* ca. 40 mm diam, subconical to convex; surface dry, covered with yellowish brown (6C6–7), light brown (6D4–5) to dark brown (6E5) or dark grey (6E1–3), more or less radially arranged, repent, fibrillose squamules; margin with ciliate squamules derived from breaking up of the veil, and the color is slightly lighter than surface of pileus; volval remnants of host present on the disc, grey. *Lamellae* white (1A1), adnexed to adnate, moderately crowded, denticulate. *Stipe* 30 *×* 6–10 mm, nearly cylindric, usually tapering upward; surface densely covered by squamules arranged in irregular fibrillose annular zone at the upper part of the stipe, extreme apex white (1A1) and nearly smooth, the part below the ring is covered with orange (6A6–7), tawny yellow (6C7) or yellowish brown (6D7–8) appressed or erect, obliquely lacerate scales. *Mycocecidium* subglobose 35 *×* 20–30 mm, nearly smooth, and whitish or grey spots on external surface. The transitional zone between stem and mycocecidium with some irregular rings of tawny-ochraceous (6B7–8) or dingy brown (6E5) color, fibrillose, appressed, or with erect, obliquely upward-pointing scales or lacerate scales.

*Basidiospores* [50/1/1] (5–) 5.5–6 (− 6.5) × 4–5 (− 6) μm [Q = (1.2–) 1.5–1.65, Q = 1.43 ± 0.10], broadly ellipsoid, ellipsoid to elongate, sometimes subreniform in side view. *Basidia* 20–35 × 5–10 μm, subclavate, 4-spored, fusiform to ventricose-fusiform, hyaline; sterigmata 3–4 μm long; basal septa often with clamps. *Cystidia* numerous, 90–105 × 17–27 μm, fusiform to ventricose-fusiform, with obtuse to acute apex, upper part slightly to moderately thick-walled (up to 1 μm diam.), sometimes with refractive incrustations, hyaline. *Lamellar trama* regular, composed of colorless, thin-walled hyphae 4–15 μm diam, branching, sometimes anastomosing; clamps present and common. *Subhymenium* 10–15 μm thick, composed of 4–6 μm wide filamentous hyphal segments; v*olval remnants* of host on pileus composed of ± irregularly arranged elements: inflated cells very abundant (to locally dominant), subglobose (30–50 × 30–50 μm) or ovoid to broadly clavate (30–60 × 20–30 μm), solitary and terminal, or in chains of 2–3 and then terminal, inflated cells sometimes external upset (up to 1 μm thick), usually colorless and hyaline, occasionally with brownish vacuolar pigments, and the majority of hyphae without clamp connection; inner part of volval remnants near pileus surface composed of ± irregularly arranged elements: inflated cells usually brownish to fawn colored, two types of filamentous hyphae in the tissues: either with filamentous hyphae usually colorless and hyaline, 2–6 μm wide, without clamp connection; or with hyphae similar to lotus root, 60–150 × 4–15 μm, swollen in the middle but constricted at septa, with clamp connection. *Mycocecidium* composed of abundant ovoid to subglobose inflated cells (45–110 × 24–65 μm) and filamentous hyphae colorless and hyaline, 2–6 μm wide, with clamp connections similar to those on the pileus; chlamydospores not observed.

*Ecology*: Parasitic on *Amanita sepiacea* (HKAS74862B, GenBank Acc. nos.: ITS = MW258870) growing on soil under trees of *Fagaceae* and *Rhododendron*.

*Distribution*: Currently known from Yunnan Province, Southwest China.

*Notes*: Our morphological data and molecular phylogenetic evidences confirm that the host of *S. orientalis* and the collection of *Amanita* in the nearby area within two kilometers’ range of *S. orientalis* are *A*. *sepiacea* (Figs. 5, 8, 10). Interestingly, some volval remnants of *A*. *sepiacea* are found on the center of the pileal surface of *S. orientalis* (Fig. 8), and its anatomical features are those of *A*. *sepiacea* (Yang 2005) (Fig. 10), and the filamentous hyphae with clamp connection belong to *S. orientalis* (Fig. 10).

*Squamanita orientalis* is similar to *S. schreieri*. However, the latter species has no cystidia. Furthermore, the former is a parasite on *A*. *sepiacea*, while *S. schreieri* is possibly associated with *A. strobiliformis* or *A. echinocephala* (Bas 1965).

*Squamanita orientalis* is also similar to *S. sororcula* and *S. umbonata*. However, *S. orientalis* differs from *S. sororcula* by its irregular fibrillose annular zone on the upper part of the stipe and ciliate squamules on the pileal margin, and larger cystidia (90–105 × 17–27 μm). In addition, there are ca. 50 and ca. 40 base differences in ITS and nrLSU regions between the two species respectively, and even though their hosts are identified as *A*. *sepiacea* for both species, there are ca. 25 different bases in the ITS region from host material. *Squamanita umbonata* differs from *S. orientalis* by its umbonate pileus, and narrower cystidia (60–95 × 9–20 μm), cylindrical to clavate fusiform mycocecidia.

**Fig. 11:**
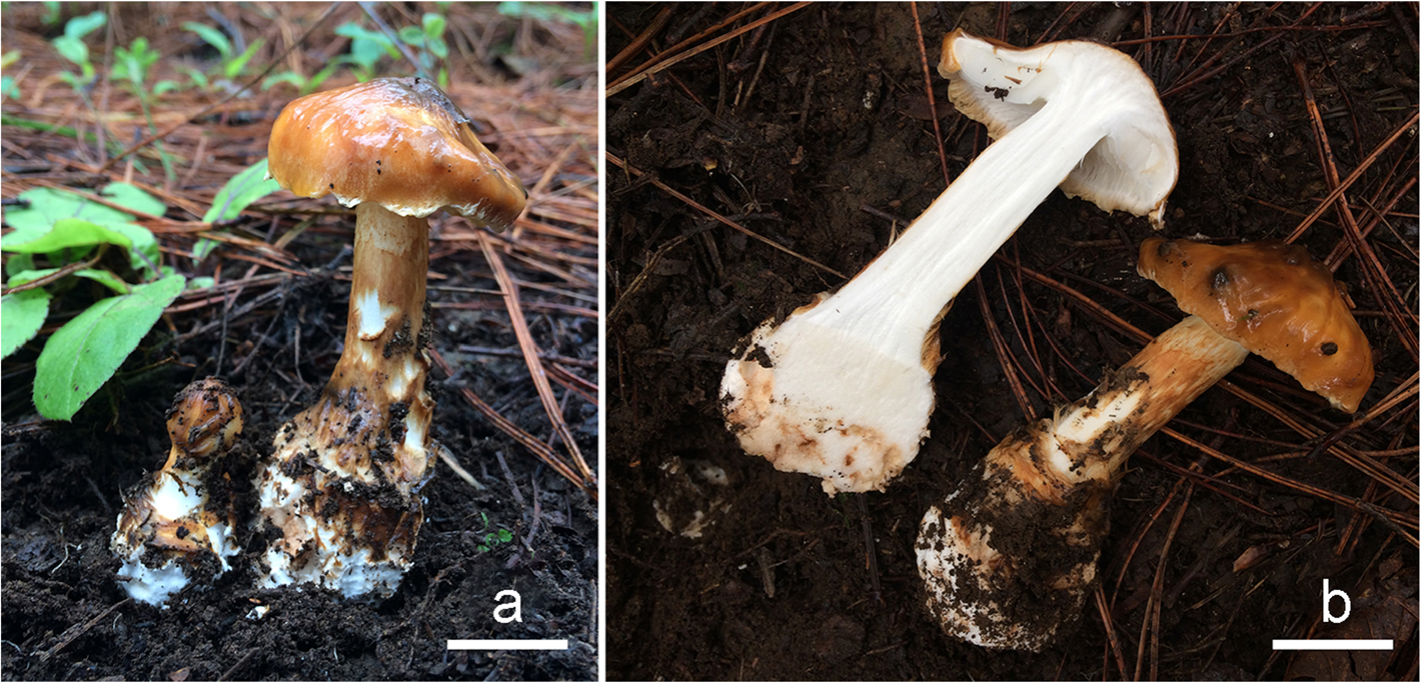
**a**–**b** Basidiomes of *Squamanita sororcula* (HKAS107306A, holotype). Photos by Fa Li. Bars: 20 mm

**Fig. 12:**
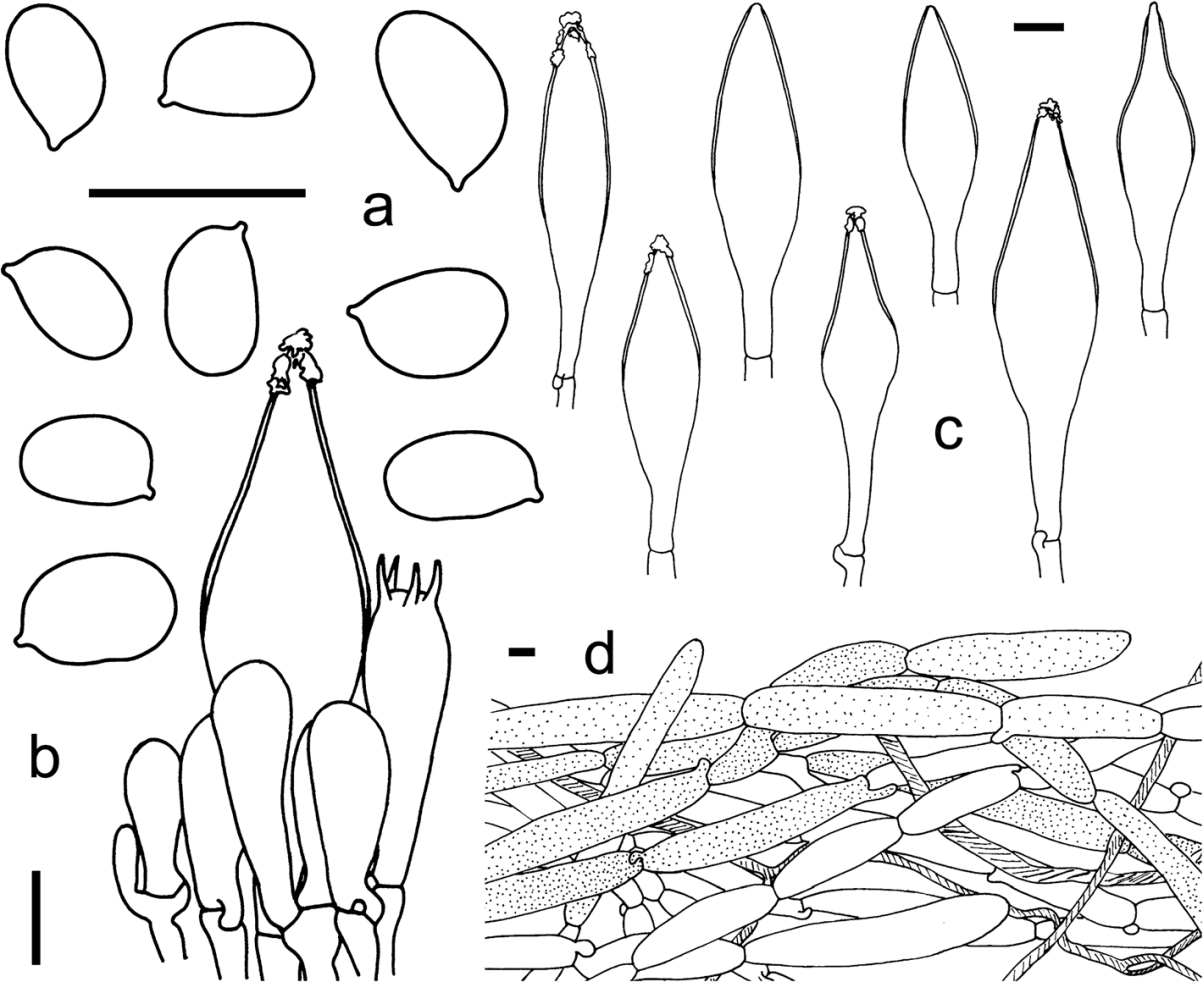
Microscopic features of *Squamanita sororcula* (HKAS107306A, holotype). **a** Basidiospores. **b** Hymenium and subhymenium with one pleurocystidia covered with refractive incrustations. **c** Pleurocystidia, four of them covered with refractive incrustations. **d** Pileipellis section. Drawings by Jianwei Liu. Bars = 10 μm

***Squamanita sororcula*** J. W. Liu & Zhu L. Yang, ***sp. nov.*** — *Fungal Names* FN570782;

MycoBank 836586. (Figs. 11 and 12).

*Etymology*: —*sororcula* (Lat.): little sister, indicating a close relation with *S. orientalis*.

*Diagnosis*: *S. sororcula* differs from other species by without irregular fibrillose annular zone on the upper part of the stipe, with subglobose mycocecidia.

*Type*: **China**: *Yunnan Province*: Laojun Mountain, Jianchuan City, Dali, 26°38′51.792″N, 99°49′10.43E, 2756 m elev., in a forest dominated by plants of *Pinus yunnanensis*, 10 Aug. 2019, *Fa Li 237* (HKAS107306A – holotype; GenBank Acc. nos.: 18S = MW258929, ITS = MW258850, nrLSU = MW258902, TEF1-α *=* MW324507).

*Description*: *Pileus* medium-sized, ca. 45 mm diam, at first globose, then hemispheric, plano-convex with slightly incurved margin, thick-fleshed; surface buff (6B7–8), viscid when wet, covered with buff (6B7–8) floccose-fibrillose or slightly fibrillose squamules; pileal margin strongly appendiculate, with irregularly and densely corniform and fibrillose squamules derived from breaking up of the veil. *Lamellae* white (1A1), adnexed to adnate, moderately crowded, denticulate, rather thin, 6–7 mm wide. *Stipe* 50 × 10–19 mm, nearly cylindric, usually tapering upward; surface covered with buff (6B7–8) floccose-fibrillose or slightly fibrillose scaly zones, but not forming an irregular fibrillose annular zone at the upper part of the stipe, extreme apex white and nearly smooth. *Mycocecidia* subglobose 35 × 25 mm, white (1A1) with brownish (6A4–5) to rusty (6B7–8) spots. The transitional zone between stem and mycocecidia with some irregular rings of tawny-ochraceous (6B7–8) or dingy brown (6E5), fibrillose, appressed, or erect, obliquely upward-pointing or lacerate scales. Context white (1A1), rather firm. Smell rather strongly musty when crushed.

*Basidiospores* [40/1/1] 5.5–7.5 (− 9) × (3.5–) 4–5 (− 5.5) μm [Q = (1.2–) 1.3–1.8 (− 2), Q = 1.6 ± 0.8], broadly ellipsoid, ellipsoid to elongate, sometimes subreniform in side view. *Basidia* 20–35 × 8–10 μm, subclavate, 4-spored, fusiform to ventricose-fusiform, hyaline; sterigmata 4–5 μm long; basal septa often with clamps. *Pleurocystidia* and *cheilocystidia* numerous, 60–90 × 13–17 μm, fusiform to ventricose-fusiform, with obtuse to acute apex, nearly all upper part of cystidia are slightly thick-walled (up to 1.5 μm), sometimes with refractive incrustations, hyaline. *Lamellar trama* regular, composed of colorless, thin-walled hyphae 5–10 μm diam, branching, sometimes anastomosing; clamps present and common. *Subhymenium* consisting of 4–6 μm wide filamentous hyphal segments, narrow. *Pileipellis* a cutis with transition to a trichoderm at regular intervals, composed of loosely and more or less radially arranged, thin-walled hyphae 60–120 × 5–20 μm, and at the upper of the pileipellis often with fine brownish granular incrustations on the yellowish to brownish filamentous hyphae, clamps present and common, occasionally with brown vacuolar pigments, 2–5 μm wide; *Mycocecidia* composed of abundant subglobose to broadly clavate inflated cells (20–55 × 20–40 μm), and colorless and hyaline clampless filamentous hyphae, 2–6 μm wide, and clamped filamentous hyphae nearly 5–15 μm wide similar to those on the pileus; chlamydospores not observed.

*Ecology*: Parastic on *Amanita sepiacea* (HKAS107306B, ITS = MW258871, TEF1-α *=* MW324505) growing on soil in forest dominated by *Pinus yunnanensis*.

*Distribution*: Currently known from Hunan and Yunnan Provinces, central and Southwest China.

*Notes*: *Squamanita sororcula* is similar to *S. mira*, *S. orientalis*, *S. schreieri*, *S. umbonata*, and other collections assigned to the “*S. umbonata*” complex. The differences between the first two and *S. sororcula* have been discussed above. Besides, *S. sororcula* differs from *S*. *schreieri* by the presence of cystidia and differs from *S. umbonata* by its subglobose mycocecidia.

Wang and Yang (2004) treated two collections (HKAS38127 and 38149) as “*S. umbonata*” collected from Hunan province, central China. Unfortunately, the collections have not been traced by us. However, the two collections are without an annular zone, and should be close to *S. sororcula* rather than *S. orientalis.*

***Key to Squamanita worldwide***

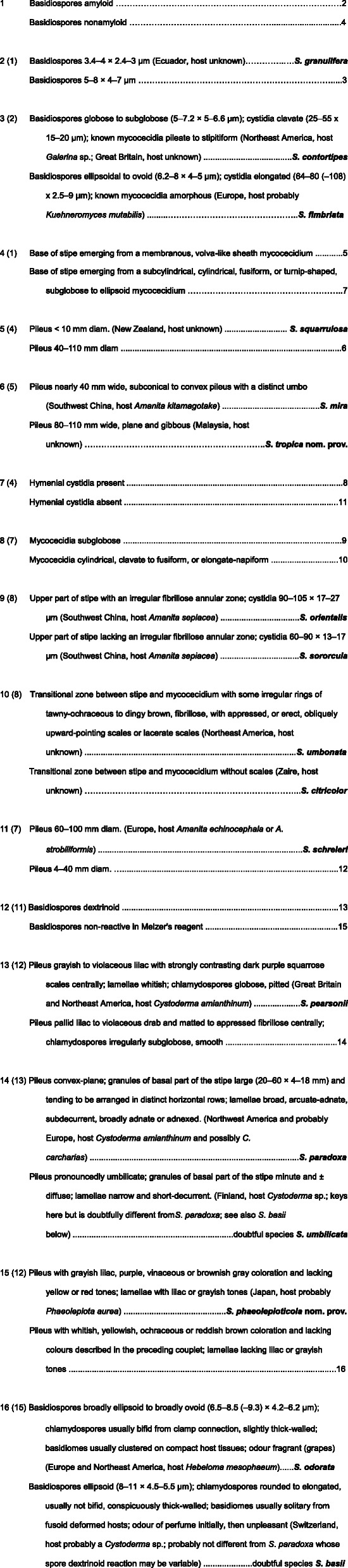



## DISCUSSION

### Systematic position of *Cystodermateae*

Singer (1986) included in *Cystodermateae* the following seven genera, viz. *Cystoderma*, *Dissoderma* (current name *Squamanita*), *Horakia* (current name *Verrucospora*), *Phaeolepiota*, *Pseudobaeospora*, *Ripartitella*, and *Squamanita*. Based on the phylogenetic analyses of Matheny and Griffith (2010), Matheny et al. (2015), Vizzini et al. (2019), Kalichman et al. (2020) and our present studies, three genera among *Cystodermateae*, viz. *Cystoderma*, *Phaeolepiota*, and *Squamanita* together with *Leucopholiota* and *Floccularia* can be assigned to the *Squamanitaceae* within *Agaricineae* (agaricoid clade). *Pseudobaeospora* was recognized as a member of the *Tricholomataceae s. str*. within *Tricholomatineae* (tricholomatoid clade) in the multigene phylogenetic analyses of Sánchez-García and Matheny (2017) and He et al. (2019). Molecular data from a species of *Verrucospora, V. flavofusca,* confirm placement in *Agaricaceae s.lat.* with strong statistic support (SH-aLRT/UFB/PP = 95.4/98/0.99) in our study (Fig. 2). Oberwinkler (1976) and Singer (1986) supposed that *Horakia* (now included in *Verrucospora*) belonged to *Thelephorales* or *Cystodermateae* of *Agaricales*, respectively, which are incorrect placements based on our molecular phylogenetic data. Phylogenetic placements of *Ripartitella*, and *Cystodermella*, which was separated from *Cystoderma* by Harmaja (2002), are unclear at present, although previous research based on nLSU, RPB1 and ITS molecular sequences indicated that *Ripartitella* and *Cystodermella* are near *Cercopemyces* (Baroni et al. 2014). Our study (Fig. 2) is consistent with Baroni et al. (2014), and these three genera are close to *Hydnangiaceae* in our phylogenetic tree (Fig. 2).

Saar et al. (2016) treated *Phaeolepiota aurea* as *Cystoderma aureum* because it was nested within *Cystoderma*. However, *P. aurea*, with large inamyloid fusoid and asperulate spores, differs from *Cystoderma*, species of which have amyloid, ellipsoid, oblong or fusiform and smooth spores. In our multigene phylogenetic tree (Fig. 2), and the supplementary trees of Varga et al. (2019), *P. aurea* nested within *Cystoderma*, but clustered with *Cystoderma superbum* (Fig. 2), a unique species commonly reported to be amyloid but in only a small area of the basidiospore surface, which is a morphotaxonomic character that differs from other species of *Cystoderma*. In the study of Matheny and Griffith (2010), and supplementary trees of that study (Additional files 1 and 2), a close relationship among *P. aurea*, *Cystoderma* and *C. superbum* was not well supported. Therefore, for the moment, we continue to recognize *Phaeolepiota* for *P. aurea*. Further studies with more samples and using more DNA makers are necessary to clarify the position of *P. aurea* and *C. superbum* in relation to other species of *Cystoderma*.

Up to now, 12 described species of *Squamanita* have been accepted, although Matheny and Griffith (2010: Table 1) listed 15, including three not validly published designations: *S*. *cettoiana* (nom. inval.), *S*. *phaelepioticola* (nom. prov.), and *S. tropica* (nom. prov.).

### Diversity of the “*S. umbonata*” species complex

**Fig. 13:**
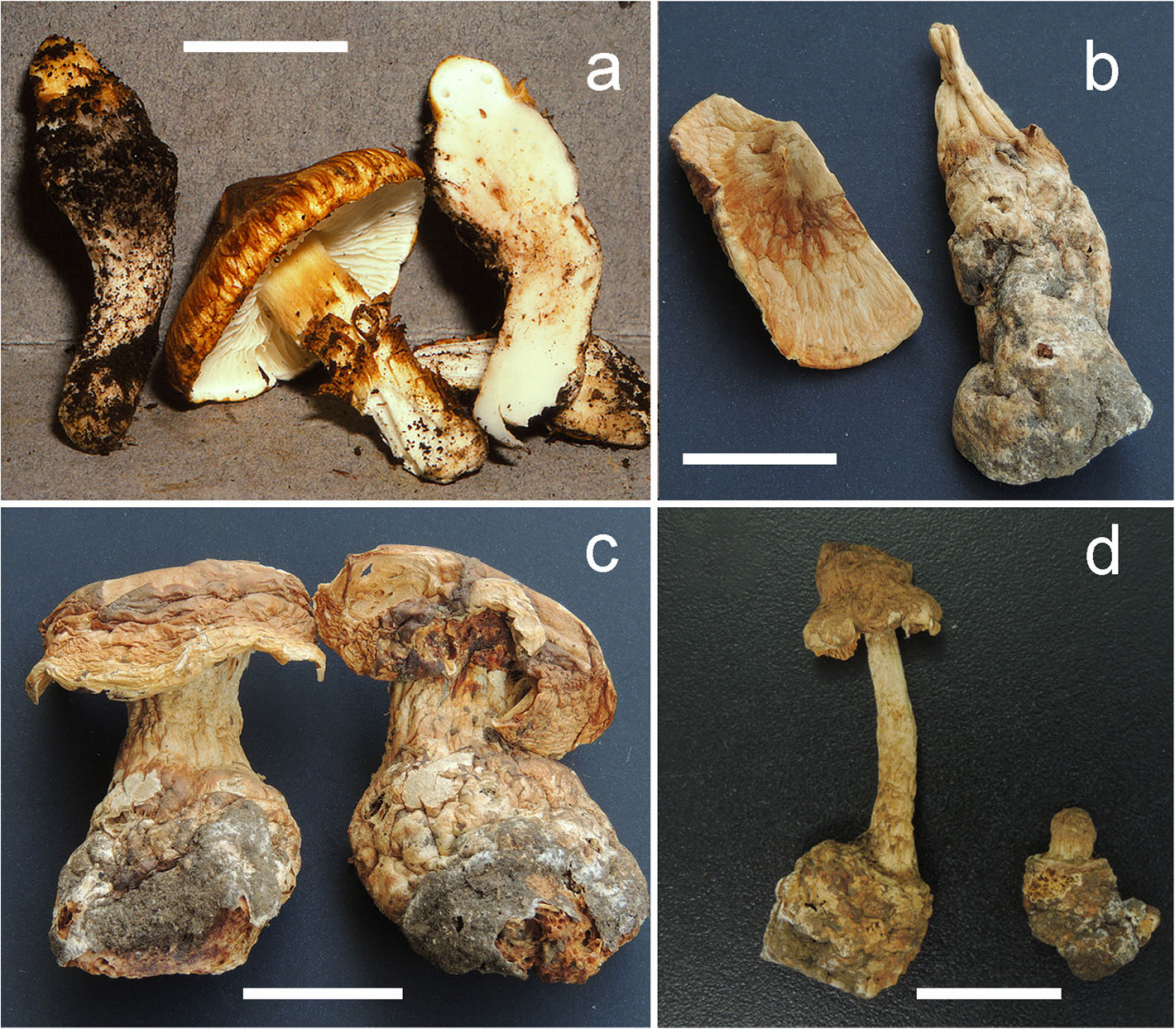
Specimens of “*Squamanita umbonata*” included in this study. **a** Fresh basidiomes of R. E. Halling 7691 with cylindrical to clavate fusiform mycocecidium from Costa Rica. **b** C. Bas 3808 from USA with cylindrical mycocecidium. **c** H. E. Bigelow 17431 from USA with subglobose mycocecidium, a lump of clay is attached on the center of pileus of the specimen on the right, and the apical part of volval remnants on mycocecidium can be observed between clay and pileus under anatomical lens. **d** HKAS107325A from Italy with subglobose mycocecidium. Bars **a** = 25 mm, **b**–**d** = 20 mm

Our study indicated that material of “*Squamanita umbonata*” from the Northern Hemisphere clustered into two species complexes each consisting of several different species (Figs. 2 and 3), including *S. orientalis*, *S. sororcula*, and several undefined specimens*.* Morphological characteristics of collection *R. E. Halling 7691* (NY79971) (Fig. 13) from Costa Rica are mostly consistent with the descriptions of the type (NY27684) by Sumstine (1914) and Bas (1965), with an umbonate pileus, cylindrical to clavate fusiform mycocecidia, and thin-walled cystidia. However, considering that the type of *S. umbonata* was from Pennsylvania, USA, we are reluctant to identify *R. E. Halling 7691* as *S. umbonata* until molecular data from the type are available.

The collection *H. E. Bigelow 17431* (NY2776224) (Fig. 13) has a subglobose mycocecidium, slightly smaller basidiospores (5–7 × 3.5–5 μm) and cystidia (45–65 × 12–18 μm) in comparison with those of *S. umbonata*, and the mycocecidium is composed of abundant inflated cells, indicating the possibility of *Amanita* as host. *C. Bas 3808* (NY1840398) (Fig. 13) was published as *S. umbonata* by Bas (1965). However, Cortés-Pérez et al. (2014) showed that the upper parts of the cystidia in this collection were slightly to moderately thick-walled, which is consistent with our observations of the collection. Phylogenetically *C. Bas 3808* forms a monophyletic branch with DAOM 199323 [GenBank accession no.: AF261508], submitted by Moncalvo et al. (2002), and may well be conspecific with that (Fig. 3). A collection from Italy ((HKAS107306A; Fig. 13) is sister to *H. E. Bigelow 17,431, C. Bas 3808*, and *DAOM 199323* (Fig. 3), and its hosts belong to the species complex of *A. excelsa* (GenBank accession no.: MW258872 and MW258873). *Squamanita umbonata* is also reported from Japan (Ikeda 1996), Italy (Vizzini and Girlanda 1997), and Mexico (Cortés-Pérez et al. 2014). Further efforts are necessary to reveal the species diversity of “*S. umbonata*” globally and delimit the constituent species, including a clear application of the name *S. umbonata*.

### Host preference or specificity of *Squamanita* species

Our study reveals that the basidiomes of *S. mira* are composed of its own hyphae, while the mycocecidia also include hyphae of the host, which is consistent with the observations on *S. paradoxa* by Mondiet et al. (2007) and Griffith et al. (2019). Interestingly, host hyphae are found in the volval remnants that are attached to the pileal surface of *S. orientalis* (Figs. 5, 8, 10). This character may provide additional help for the host identification of *Squamanita*. Although sometimes the basidiomes of *Squamanita* may macromorphologically deform the hosts, most of the time the shapes of infected hosts (mycocecidia) still largely maintain consistent morphological characteristics with nearby uninfected basdiomes of the same species. Our study showed that *S. orientalis*, *S. sororcula* and “*S. umbonata*” (HKAS107325A) from Italy, with subglobose mycocecidia, are parasitic on *A.* sect. *Validae*, while *S. mira*, with the sheathing volva arising from the margin of a bulb, is parasitic on *A*. *kitamagotake*. Therefore, the shape and the size of the mycocecidia could be a reliable morphological character at species level.

## CONCLUSION

The monophyly of the family *Squamanitaceae* was confirmed by multi-gene Bayesian phylogenetic analysis, with five genera, namely *Cystoderma*, *Phaeolepiota*, *Squamanita*, *Floccularia* and *Leucopholiota* falling in the family. Three new species from China, parasitizing two different species from two sections of *Amanita*, were uncovered and described based on morphological and molecular evidence. Furthermore, a multi-gene phylogenetic analysis on “*Squamanita umbonata*” from North America, Central America, Europe, and East Asia showed that it represents two species complexes harboring eight subclades. Further morphological studies are needed to reveal the species diversity and distribution patterns of “*Squamanita umbonata*”.

## Supplementary Information

**Additional file 1. **Maximum-Likelihood (ML) phylogenetic tree of *Squamanitaceae* inferred from ITS sequences, with SH-aLRT (left), ultrafast bootstrap (UFB) (right), only one of SH-aLRT > 80 or UFB > 95 for ML are indicated along branches (SH-aLRT/UFB). New species *Squamanita mira*, *S. orientalis*, *S. sororcula* are highlighted in boldface.

**Additional file 2. **Maximum-Likelihood (ML) phylogenetic tree of *Squamanitaceae* inferred from LSU sequences, with SH-aLRT (left), ultrafast bootstrap (UFB) (right), only one of SH-aLRT > 80 or UFB > 95 for ML are indicated along branches (SH-aLRT/UFB). New species *Squamanita mira*, *S. orientalis*, *S. sororcula* are highlighted in boldface.

## Data Availability

The datasets generated for this study (Tables 1 and 2) can be accessed via GenBank: https://www.ncbi.nlm.nih.gov/genbank/. Alignments analysed during the current study are available at TreeBase: https://www.treebase.org/.

## Abbreviations

nrLSU: The large nuclear ribosomal RNA subunit
ITS: The internal transcribed spacers 1 and 2 with the 5.8S rDNA
5.8S: 5.8S gene
18S: The small subunit region
TEF1*-*α: Translation elongation factor 1-α
RPB1: RNA polymerase II largest subunit
RPB2: RNA polymerase II second largest subunit
CTAB: Cetyltrimethyl ammonium bromide
HKAS: Herbarium of Cryptogams, Kunming Institute of Botany of the Chinese Academy of Sciences
ML: Maximum likelihood
UFB: Ultrafast bootstrap support values of IQTREE
SH-aLRT: The Shimodaira–Hasegawa-like aLRT test support values
PP: Bayesian posterior probability
NY: The New York Botanical Garden
