# Supplementary figures and images for: Squamanitaceae and three new species of Squamanita parasitic on Amanita basidiomes
Source: IMA Fungus. 2021 Mar 3;12:4. doi: 10.1186/s43008-021-00057-z (PMC7927255; doi:10.1186/s43008-021-00057-z)

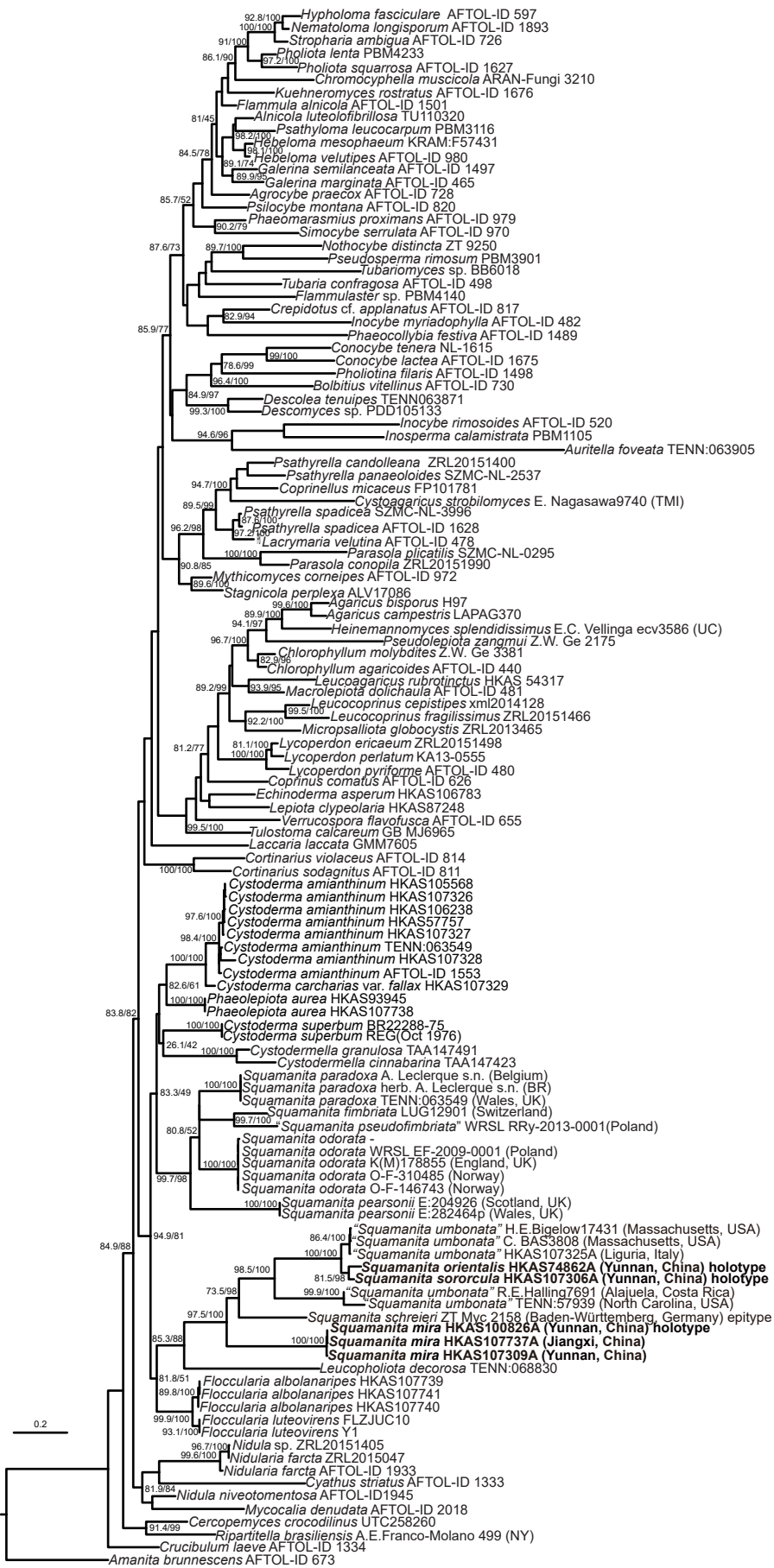

Supplement: Supplementary file 1 — Additional file 1. Maximum-Likelihood (ML) phylogenetic tree of Squamanitaceae inferred from ITS sequences, with SH-aLRT (left), ultrafast bootstrap (UFB) (right), only one of SH-aLRT > 80 or UFB > 95 for ML are indicated along branches (SH-aLRT/UFB). New species Squamanita mira, S. orientalis, S. sororcula are highlighted in boldface. [file 43008_2021_57_MOESM1_ESM.pdf]

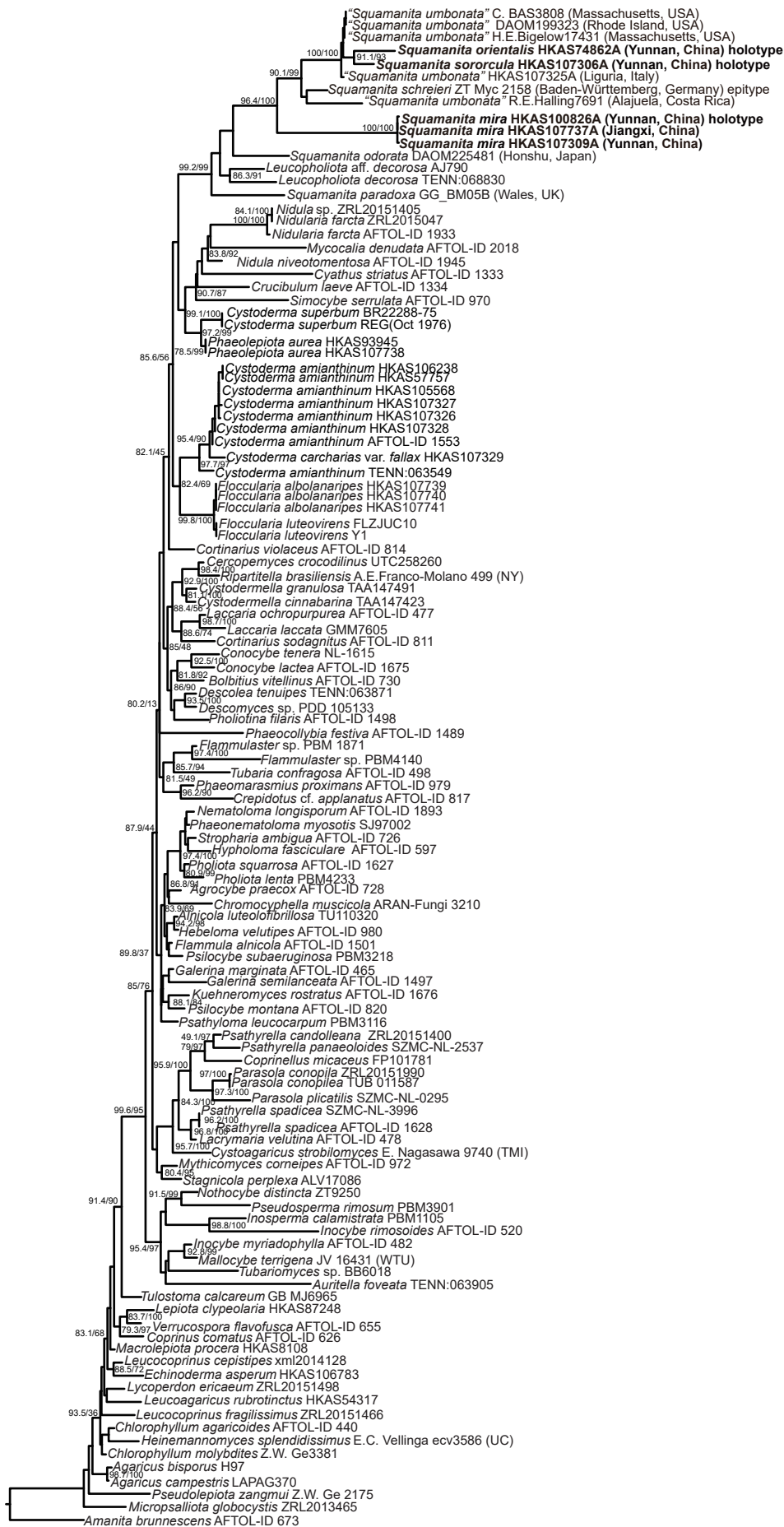

0.04

Supplement: Supplementary file 2 — Additional file 2. Maximum-Likelihood (ML) phylogenetic tree of Squamanitaceae inferred from LSU sequences, with SH-aLRT (left), ultrafast bootstrap (UFB) (right), only one of SH-aLRT > 80 or UFB > 95 for ML are indicated along branches (SH-aLRT/UFB). New species Squamanita mira, S. orientalis, S. sororcula are highlighted in boldface. [file 43008_2021_57_MOESM2_ESM.pdf]
